# Real-world safety and effectiveness of nivolumab in Japanese patients with unresectable advanced or recurrent gastric/gastroesophageal junction cancer that has progressed after chemotherapy: a postmarketing surveillance study

**DOI:** 10.1007/s10120-021-01244-y

**Published:** 2021-09-28

**Authors:** Kensei Yamaguchi, Narikazu Boku, Kei Muro, Kazuhiro Yoshida, Hideo Baba, Shinji Tanaka, Ayumi Akamatsu, Takeshi Sano

**Affiliations:** 1 Department of Gastroenterological Chemotherapy, Cancer Institute Hospital of Japanese Foundation for Cancer Research, 3-8-31 Ariake, Koto-Ku, Tokyo, 135-8550 Japan; 2 Department of Gastrointestinal Medical Oncology, National Cancer Center Hospital, Tokyo, Japan; 3 Department of Clinical Oncology, Aichi Cancer Center, Nagoya, Japan; 4 Department of Surgical Oncology, Gifu University Graduate School of Medicine, Gifu, Japan; 5 Department of Gastroenterological Surgery, Graduate School of Medical Sciences, Kumamoto University, Kumamoto, Japan; 6 Pharmacovigilance Division, Ono Pharmaceutical Co., Ltd, Osaka, Japan; 7 Department of Gastroenterological Surgery, Cancer Institute Hospital of Japanese Foundation for Cancer Research, Tokyo, Japan

**Keywords:** Real world, Postmarketing surveillance, Gastric or gastroesophageal junction cancer, Nivolumab, Japan

## Abstract

**Background:**

This postmarketing surveillance study evaluated the real-world safety and effectiveness of nivolumab as salvage (after ≥ 2 lines) therapy in Japanese patients with unresectable advanced or recurrent gastric/gastroesophageal junction (G/GEJ) cancer.

**Methods:**

This multicenter, observational study was conducted at 158 centers in Japan. Patients with unresectable advanced or recurrent G/GEJ cancer were registered between Nov 1, 2017, and Oct 31, 2018, and observed for 6 months after treatment initiation with nivolumab. Correlation of background characteristics with treatment-related adverse events (TRAEs) and tumor response was explored.

**Results:**

Overall, 654 patients were registered (safety analysis set, *n* = 650; effectiveness analysis set, *n* = 636; response evaluation set, *n* = 516). The incidences of all TRAEs and grade ≥ 3 TRAEs were 31.5 and 11.2%, respectively. TRAEs significantly correlated with the absence of peritoneal metastasis; C-reactive protein level < 1; prior G/GEJ cancer surgery; and past or concomitant pulmonary, thyroid, or renal disease (each *p* < 0.05). The incidence of TRAEs was significantly lower in patients with higher Glasgow prognostic scores (*p* < 0.05). No new safety signals were observed. Complete response, partial response, stable disease, and progressive disease were observed in 1.2, 10.1, 27.1, and 58.3% of the response evaluation set, respectively. Patients aged ≥ 65 years (13.9 vs 5.3%, *p* = 0.0083) and ≥ 75 years (18.8 vs 9.2%, *p* = 0.0036) showed a higher response rate than their younger counterparts.

**Conclusions:**

The real-world safety and effectiveness of nivolumab as salvage (after ≥ 2 lines) therapy in Japanese patients with unresectable advanced or recurrent G/GEJ cancer were consistent with those observed in the phase 3 ATTRACTION-2 study.

**Supplementary Information:**

The online version contains supplementary material available at 10.1007/s10120-021-01244-y.

## Background

Gastric cancer is the fifth most common cancer and the third leading cause of cancer-related deaths worldwide [1]. Gastric/gastroesophageal junction (G/GEJ) cancer accounts for ~ 783,000 deaths, with over 1,000,000 new cases of G/GEJ cancer reported worldwide in 2018 [1]. The cumulative risk of developing gastric cancer from birth to age 74 years is higher in men (1.87%) than in women (0.79%) [1]. In Japan, gastric cancer was the third most common cause of cancer-related deaths in 2018 [2].

Nivolumab, an immune checkpoint inhibitor and a fully humanized anti-programmed cell death protein 1 (PD-1) monoclonal antibody, has been approved for various indications in more than 65 countries, including the United States, European Union, and countries in Asia [3]. In the ATTRACTION-2 study, nivolumab, when compared with placebo in patients with unresectable advanced or recurrent G/GEJ cancer after failure of ≥ 2 lines of chemotherapy [4], improved the overall survival (OS; median, 5.26 vs 4.14 months), with a better 1-year OS rate (26.2 vs 10.9%) [4]. Consequently, the results of ATTRACTION-2 [4] led to the approval of nivolumab as a third- or later-line therapeutic option for patients with unresectable advanced or recurrent G/GEJ cancer in several countries, including Japan [5]. In Japan and Korea, nivolumab is recommended as third- or later-line therapy in the treatment guidelines of gastric cancer [5, 6].

However, as ATTRACTION-2 was limited by its sample size and stringent inclusion/exclusion criteria, the results from this study do not sufficiently reflect the real-world scenario, where not a few patients to be excluded from the clinical trials are treated with nivolumab. Accordingly, the Ministry of Health, Labour and Welfare (MHLW) required a postmarketing surveillance (PMS) study after drug approval. Therefore, this PMS study was conducted in Japan to evaluate the real-world safety and effectiveness of nivolumab in patients with unresectable advanced or recurrent G/GEJ cancer having diverse background factors. To the best of our knowledge, this is the first real-world evaluation of an immune checkpoint inhibitor in over 600 Japanese patients with unresectable advanced or recurrent G/GEJ cancer.

## Methods

This PMS was a multicenter, open-label, observational study conducted at 158 centers in Japan. Patients with unresectable advanced or recurrent G/GEJ cancer to be treated with nivolumab (*n* = 654) were registered between Nov 1, 2017, and Oct 31, 2018. These registered patients were observed for 6 months or until death after treatment initiation with nivolumab.

### Patients

Patients with unresectable advanced or recurrent G/GEJ cancer who newly received nivolumab after failure of ≥ 2 lines of chemotherapy were registered for this PMS study. Patients were excluded from the effectiveness analysis if they received nivolumab as off-label use (treatment for an indication other than unresectable advanced or recurrent G/GEJ cancer after failure of ≥ 2 lines of chemotherapy before the initiation of nivolumab).

### Treatment

Nivolumab at the approved dose as described in the prescribing information was administered. Of note, the approved dose was changed from the body weight–equivalent dose (3 mg/kg) to a fixed dose of 240 mg/body that received marketing authorization in August 2018. Treatment was discontinued based on the physician’s discretion owing to progressive disease (PD) according to the Response Evaluation Criteria in Solid Tumors (RECIST) v1.1, lack of effectiveness even if the criteria for PD were not met, intolerability, or patients’ refusal.

### Safety

Adverse events (AEs) and treatment-related adverse events (TRAEs) were reported; their relation to nivolumab was judged by each attending physician. The clinical course of TRAEs of special interest was monitored. All AEs were graded using the National Cancer Institute Common Terminology Criteria for Adverse Events (NCI CTCAE v4.0 or v5.0).

### Effectiveness

The question, “Was the response evaluated in accordance with the RECIST v1.1?,” was included in the case report form (CRF). If the answer was “Yes,” patients were included in the response evaluation set, in which tumor response [complete response (CR), partial response (PR), stable disease (SD), and PD] was assessed, and the objective response rate (ORR; CR or PR) was obtained. The 6-month survival rate was evaluated using the Kaplan–Meier method.

### Statistical analysis

The sample size of the safety analysis set was planned at 500, which provided a power of 77.74% to detect ≥ 1 AE with a frequency of 0.30%, corresponding to the lowest frequency of AEs that occurred in the ATTRACTION-2 trial (0.30%; 1/330 patients).

Exploratory subgroup analyses were performed on the incidence of TRAEs and the ORR; the comparison between subgroups was performed using Fisher’s exact test, Wilcoxon rank sum test, or chi-squared test. Association between each patient characteristics, excluding the factors which allowed double count such as tumor location and histological differentiation, and the incidences of TRAEs and ORR were analyzed using multivariate logistic regression to adjust the number of nivolumab doses (≥ 5 vs ≤ 4).

## Results

### Patient disposition, demographics, and baseline characteristics

A total of 654 patients were registered, of whom 650 comprised the safety analysis set and 636 comprised the effectiveness analysis set. Patient disposition is shown in Supplementary Fig. 1. Patient demographics and baseline characteristics of the safety analysis set are shown in Table 1. In the safety analysis set, 73% of patients were men; the median (range) age was 69 (20–87) years; 90% of patients had an Eastern Cooperative Oncology Group performance status (ECOG PS) of 0 or 1; and the median number of prior treatment regimens was 2 (0–6). The number of patients with poorly differentiated adenocarcinoma and well-differentiated adenocarcinoma was 385 and 343, respectively, allowing a double count if a patient had histological components of both types. In addition, the proportion of patients with a large amount of ascites was 17.7% and those with past or present comorbidities was 65.8%.

**Table 1:** Patient demographics and baseline characteristics (safety analysis set)

	Patients, *n* (%) *N* = 650
Age (1) (years)	
15 to < 65	183 (28.2)
65 to < 75	301 (46.3)
≥ 75	166 (25.5)
Age (2) (years)	
≥ 65	467 (71.9)
Age (3) (years)	
< 75	484 (74.5)
Sex	
Male	474 (72.9)
Female	176 (27.1)
ECOG PS	
0	265 (40.8)
1	320 (49.2)
2	55 (8.5)
3	10 (1.5)
4	0 (0.0)
ECOG PS	
0–1	585 (90.0)
2–4	65 (10.00)
Smoking	
Smoker	338 (52.0)
Nonsmoker	238 (36.6)
Unknown	74 (11.4)
Any past or present comorbidity	
No	221 (34.0)
Yes	428 (65.8)
Unknown	1 (0.2)
Past or present renal disease	
No	610 (93.9)
Yes	39 (6.0)
Unknown	1 (0.2)
Past or present pulmonary disease	
No	591 (90.9)
Yes	58 (8.9)
Unknown	1 (0.2)
Past or present thyroid disease	
No	607 (93.4)
Yes	41 (6.3)
Unknown	2 (0.3)
Location^a^	
Gastroesophageal junction	94
Stomach	543
Others	8
Unknown	7
Prior surgery	
Absent	266 (40.9)
Present	384 (59.1)
Histological type^a,^^b^	
Well-differentiated adenocarcinoma	343
Poorly differentiated adenocarcinoma	385
Others	7
Unknown	4
*HER2* status	
Negative	469 (72.2)
Positive^c^	127 (19.5)
Unknown	54 (8.3)
Peritoneal metastasis	
Absent	299 (46.0)
Present	351 (54.0)
Amount of ascites	
None or small amount	528 (81.2)
Large amount	115 (17.7)
Unknown	7 (1.1)
NLR	
< 2.5	321 (49.4)
≥ 2.5	320 (49.2)
Unknown	9 (1.4)
CRP (mg/dL)	
< 1.0	431 (66.3)
> 1.0	195 (30.0)
Unknown	24 (3.7)
Glasgow prognostic score	
0	434 (66.8)
1	39 (6.0)
2	151 (23.2)
Unknown	26 (4.0)

*CRP* C-reactive protein, *ECOG PS* Eastern Cooperative Oncology Group performance status, *FISH* fluorescence in situ hybridization, *HER2* human epidermal growth factor receptor 2, *IHC* immunohistochemistry, *NLR* neutrophil to lymphocyte ratio

^a^The total number exceeds the number of the whole population because more than one category for tumor location and histological differentiation were checked in the case report form in some patients

^b^Well-differentiated adenocarcinoma includes papillary adenocarcinoma and tubular adenocarcinoma, whereas poorly differentiated adenocarcinoma includes signet ring cell carcinoma and mucinous adenocarcinoma

^c^IHC3 + or IHC2 + and FISH +

### Treatment

A total of 460 (70.8%) and 69 (10.6%) patients received nivolumab at doses of 3 and 240 mg/body intravenous infusion per cycle, respectively, throughout the study period. In 77 (11.8%) patients, the dose was changed from 3 to 240 mg/body during the study period at the physician’s discretion, while 44 (6.8%) patients received other doses. The mean ± standard deviation (median; range) number of nivolumab administration was 5.7 ± 4.1 (4.5; 1–14) in the safety analysis set.

A total of 106 (16%) patients continued treatment with nivolumab throughout the 6-month observation period. Among the remaining 544 (84%) patients, the major reasons for nivolumab discontinuation within 6 months were disease progression (including death; 71.8%), AEs (10.9%), lack of effectiveness (8.9%), and hospital transfer (1.1%). One patient (0.2%) discontinued treatment with nivolumab due to achieving a complete response as determined by the physician according to RECIST.

### Safety

**Fig. 1:**
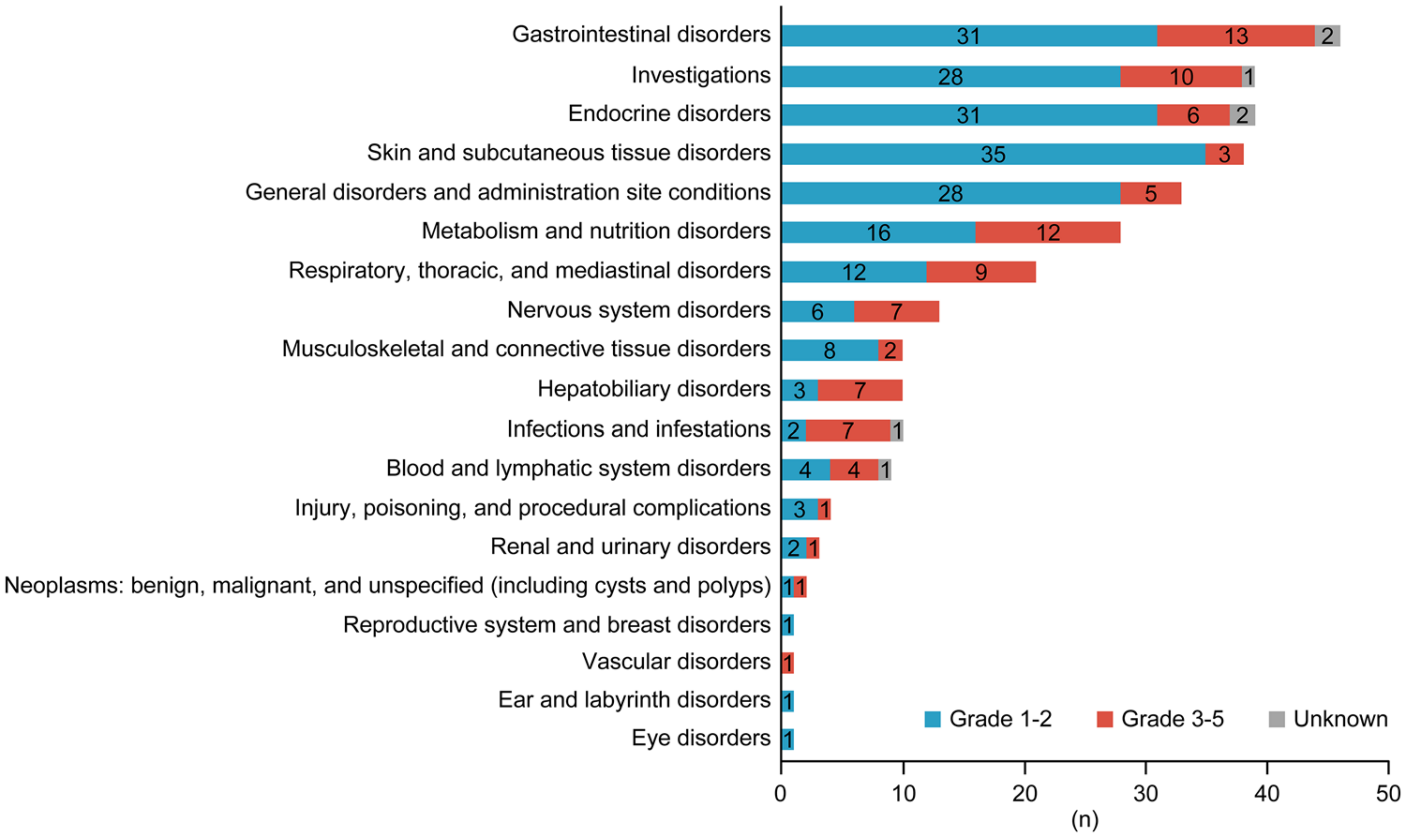
Incidence of treatment-related adverse events categorized by grade

TRAEs (all grades) were reported in 205 (31.5%) patients (Fig. 1). The median (range) duration from initiating nivolumab to occurrence of TRAEs was 32 (1–178) days. The most common TRAEs (> 2%) were hypothyroidism (4.2%), diarrhea (3.7%), decreased appetite (2.9%), rash (2.5%), and malaise (2.3%). Grade ≥ 3 TRAEs were observed in 11.2% of patients, and the most common grade ≥ 3 TRAEs were decreased appetite and diarrhea (0.9% each).

### TRAEs categorized by patient background characteristics

Incidences of TRAEs categorized by patient background characteristics are shown in Supplementary Table 1. In the univariate analysis, the following subsets showed significantly higher incidences of TRAEs: those without vs with peritoneal metastasis (35.8 vs 27.9%; *p* = 0.0344), C-reactive protein (CRP) level < 1.0 vs ≥ 1.0 (33.6 vs 25.6%; *p* = 0.0455), prior surgical treatment for G/GEJ cancer ( +) vs ( −) (34.6 vs 27.1%; *p* = 0.0482), and neutrophil to lymphocyte ratio < 5 vs ≥ 5 (33.4 vs 22.2%; *p* = 0.0281). More remarkably, a significantly higher incidence of TRAEs was observed in patients with vs without past or present comorbidities [34.8% (149/428) vs 25.3% (56/221); *p* = 0.0160], including pulmonary disease [48.3% (28/58) vs 29.9% (177/591); *p* = 0.0071], thyroid disease [48.8% (20/41) vs 30.3% (184/607); *p* = 0.0225], and renal disease [48.7% (19/39) vs 30.5% (186/610); *p* = 0.0212], and patients with lower Glasgow prognostic scores (*p* = 0.0298). TRAEs related to the kidneys were observed in none and in 0.49% (3/610) of patients with and without past or present renal disease. TRAEs related to the lungs were observed in a higher proportion of patients with [8.62% (5/58)] vs without [2.71% (16/591)] past or present pulmonary disease. TRAEs related to the endocrine system were observed in a higher proportion of patients with [26.83% (11/41)] vs without [4.45% (27/607)] past or present thyroid disease (Supplementary Table 2). Detailed information of past and present renal, pulmonary, and thyroid comorbidities categorized using system organ class and preferred terms are summarized in Supplementary Tables 3, 4, and 5. Although the incidence of TRAEs was statistically significantly associated with number of treatment lines before initiating nivolumab (*p* = 0.0281) and number of doses (*p* = 0.0337), a clear linear relationship was not observed; the incidence showed a peak at 3 treatment lines and 9–12 doses.

The multivariate analysis for incidence of TRAEs according to background factors adjusted by the number of nivolumab doses (≥ 5 vs ≤ 4) showed a similar significant increase in the incidence of TRAEs among patients with vs without peritoneal metastasis (*p* = 0.0494); and patients with any past or present comorbidities (*p* = 0.0259), including pulmonary (*p* = 0.0055), thyroid (*p* = 0.0287), and renal (*p* = 0.0189) diseases (Table 2).

**Table 2:** Multivariate analysis for the risk of TRAEs according to background factors adjusted by the number of nivolumab doses (≥ 5 vs ≤ 4 times) (safety analysis set)

Background factor^a^	Comparison	Odds ratio	95% CI	*p* value
Age (1)	65 to < 75 years vs 15 to < 65 years	1.20	0.80–1.80	0.3902
Age (2)	≥ 75 years vs 15 to < 65 years	1.44	0.91–2.28	0.1172
Age (3)	≥ 65 vs < 65 years	1.28	0.87–1.87	0.2050
Age (4)	≥ 75 vs < 75 years	1.29	0.88–1.87	0.1872
ECOG PS	2–4 vs 0–1	0.76	0.41–1.38	0.3621
Smoking history	Smoker vs nonsmoker	1.13	0.79–1.62	0.5131
Any past or present comorbidity	Yes vs no	1.52	1.05–2.19	0.0259*
Past or present renal disease	Yes vs no	2.19	1.14–4.20	0.0189*
Past or present pulmonary disease	Yes vs no	2.17	1.26–3.74	0.0055*
Past or present thyroid disease	Yes vs no	2.05	1.08–3.89	0.0287*
Peritoneal metastasis	Yes vs no	0.72	0.51–1.00	0.0494*
Prior surgery for G/GEJ cancer	Yes vs no	1.38	0.97–1.94	0.0708
Location: GEJ	Yes vs no	1.03	0.64–1.65	0.9050
Location: stomach	Yes vs no	0.98	0.63–1.53	0.9216
Location: others	Yes vs no	1.37	0.32–5.80	0.6728
Histological type: well-differentiated adenocarcinoma^b^	Yes vs no	1.28	0.92–1.79	0.1494
Histological type: poorly differentiated adenocarcinoma^c^	Yes vs no	0.82	0.59–1.15	0.2543
Histological type: others	Yes vs no	1.46	0.32–6.64	0.6221
Amount of ascites	Large vs none or small	0.70	0.44–1.11	0.1310
CRP^d^ (mg/dL)	≥ 1.0 vs < 1.0	0.74	0.50–1.10	0.1364
Glasgow prognostic score^d^ (1)	1 vs 0	0.87	0.59–1.28	0.4730
Glasgow prognostic score^d^ (2)	2 vs 0	0.65	0.40–1.06	0.0815
NLR^d^	≥ 2.5 vs < 2.5	0.81	0.57–1.13	0.2159

*CI* confidence interval, *CRP* C-reactive protein, *ECOG PS* Eastern Cooperative Oncology Group performance status, *GEJ* gastroesophageal junction, *NLR* neutrophil to lymphocyte ratio, *TRAE* treatment-related adverse event

^a^Each multivariate analysis was assessed between nivolumab doses (≥ 5 vs ≤ 4 times) and each background factor

^b^Well-differentiated adenocarcinoma includes papillary adenocarcinoma and tubular adenocarcinoma

^c^Poorly differentiated adenocarcinoma includes poorly differentiated, signet ring cell carcinoma, and mucinous adenocarcinoma

^d^Within 2 weeks before initiating nivolumab

^*^p < 0.05 was considered to be statistically significant

### Clinical course of patients with TRAEs of special interest

The clinical course of patients with TRAEs of special interest is summarized in Table 3. Among different TRAEs, the recovery rate ranged from 0 to 82.1%. Among patients who recovered, the median (range) time to recover was 100.0 (8–147) days in patients reporting thyroid dysfunction [19/35 (54%)], 22.0 (2–121) days in patients reporting colitis or severe diarrhea [23/28 (82%)], 29.0 (14–120) days in patients reporting hepatic dysfunction [13/22 (59%)], and 35.0 (17–170) days in patients reporting interstitial lung disease [11/15 (73%)]. Deaths related to TRAEs of special interest occurred in 4 patients: colitis and severe diarrhea (*n* = 1), interstitial lung disease (*n* = 2), and venous thromboembolism (*n* = 1).

**Table 3:** Outcomes of TRAEs of special interest

TRAEs of special interest	*N*	Resolved/resolving, *n* (%)	Time to recovery, (days)	Recovered with sequelae, *n* (%)	Not recovered, *n* (%)	Death, *n* (%)	Unknown, *n* (%)
			Median (min–max)				
Thyroid dysfunction	35	19 (54.3)	100.0 (8–147)	0 (0.0)	13 (37.1)	0 (0.0)	3 (8.6)
Colitis and severe diarrhea	28	23 (82.1)	22.0 (2–121)	0 (0.0)	4 (14.3)	1 (3.6)	0 (0.0)
Infusion reaction (within 24 h)	22	18 (81.8)	15.0 (1–120)	0 (0.0)	3 (13.6)	0 (0.0)	1 (4.5)
Hepatic function disorder	22	13 (59.1)	29.0 (14–120)	0 (0.0)	8 (36.4)	0 (0.0)	1 (4.6)
Interstitial lung disease	15	11 (73.3)	35.0 (17–170)	0 (0.0)	2 (13.3)	2 (13.3)	0 (0.0)
Renal disorder	6	4 (66.7)	39.5 (8–108)	0 (0.0)	2 (33.3)	0 (0.0)	0 (0.0)
Adrenal disorder	4	3 (75.0)	17.0 (14–162)	0 (0.0)	1 (25.0)	0 (0.0)	0 (0.0)
Venous thromboembolism	2	1 (50.0)	22.0 (22–22)	0 (0.0)	0 (0.0)	1 (50.0)	0 (0.0)
Type 1 diabetes mellitus	2	0 (0.0)	–	1 (50.0)	1 (50.0)	0 (0.0)	0 (0.0)
Sclerosing cholangitis	1	0 (0.0)	–	0 (0.0)	1 (100.0)	0 (0.0)	0 (0.0)
Nerve disorder	1	0 (0.0)	–	0 (0.0)	1 (100.0)	0 (0.0)	0 (0.0)
Myasthenia gravis, myocarditis, myositis, and rhabdomyolysis	1	0 (0.0)	–	1 (100.0)	0 (0.0)	0 (0.0)	0 (0.0)

*max* maximum, *min* minimum, *TRAE* treatment-related adverse event

### Effectiveness

Tumor response in the response evaluation set (*n* = 516) was CR in 6 (1.2%) patients, PR in 52 (10.1%) patients, SD in 140 (27.1%) patients, PD in 301 (58.3%) patients, and not evaluated (NE) in 17 patients (3.3%). The 6-month survival rate was 52.0%.

### ORR categorized by patient background characteristics

ORR was assessed in subgroups categorized by patient background characteristics among 499 patients in the response evaluation set (*n* = 516) after excluding 17 patients assessed as “NE”. Patients aged ≥ 65 vs < 65 years and ≥ 75 vs < 75 years showed a significantly higher ORR (*p* = 0.0083 and *p* = 0.0036, respectively) (Supplementary Table 6). The multivariate analysis for tumor response according to background factors adjusted by the number of nivolumab doses (≥ 5 vs ≤ 4) showed a significantly higher ORR among patients in the higher age group [≥ 75 years vs 15 to < 65 years (*p* = 0.0036); ≥ 65 vs < 65 years (*p* = 0.0178); ≥ 75 vs < 75 years (*p* = 0.0097)] (Table 4). Objective response was achieved in 38 (18.5%) patients who reported TRAEs [CR in 4 (2.0%) patients and PR in 34 (16.6%) patients] and in 30 (6.7%) patients without TRAEs [CR in 3 (0.7%) patients and PR in 27 (6.1%) patients].

**Table 4:** Multivariate analysis for tumor response according to background factors adjusted by the number of nivolumab doses (≥ 5 vs ≤ 4 times) (response evaluation set: excluding patients whose tumor response was NE; patients whose evaluation method complied with RECIST v1.1)

Background factor^a^	Comparison	Odds ratio	95% CI	*p* value
Age (1)	65 to < 75 years vs 15 to < 65 years	2.19	0.91–5.26	0.0810
Age (2)	≥ 75 years vs 15 to < 65 years	3.83	1.55–9.47	0.0036*
Age (3)	≥ 65 vs < 65 years	2.74	1.19–6.31	0.0178*
Age (4)	≥ 75 vs < 75 years	2.18	1.21–3.94	0.0097*
ECOG PS	2–4 vs 0–1	1.24	0.34–4.53	0.7448
Smoking history (past)	Smoker vs nonsmoker	1.86	0.99–3.47	0.0525
Amount of ascites	Large vs none or small	1.34	0.58–3.10	0.4951
NLR^b^	≥ 2.5 vs < 2.5	1.20	0.68–2.13	0.5355

*CI* confidence interval, *ECOG PS* Eastern Cooperative Oncology Group performance status, *NLR* neutrophil to lymphocyte ratio, *RECIST* Response evaluation criteria in solid tumors

^a^Each multivariate analysis was conducted between nivolumab doses (≥ 5 vs ≤ 4 times) and each background factor

^b^Within 2 weeks before initiating nivolumab

^*^p < 0.05 was considered to be statistically significant

## Discussion

This large PMS study was conducted to assess the real-world safety and effectiveness of nivolumab after failure of ≥ 2 lines of chemotherapy in Japanese patients with unresectable advanced or recurrent G/GEJ cancer. The incidence of all TRAEs and grade ≥ 3 TRAEs was 31.5 and 11.2%, respectively. The occurrence of TRAEs categorized by system organ class was similar to that reported in the ATTRACTION-2 study [4], in which TRAEs of any grade were reported in 141 (43%) patients in the nivolumab group, including 34 (10%) with grade 3–4 TRAEs [4]. Clinical trials mandate a more detailed monitoring of adverse reactions compared with PMS studies, which potentially results in slightly higher incidences compared with those observed in clinical practice.

Patients with poor medical conditions, such as ascites and comorbidities, were excluded from the ATTRACTION-2 study [4], and, therefore, the safety of nivolumab in these patients could not be evaluated. In this PMS, multivariate analysis showed that patients with past and present comorbidities such as pulmonary disease, thyroid disease, and renal disease, experienced significantly more TRAEs, suggesting that comorbidities likely affect the overall incidence of TRAEs in patients treated with nivolumab. Although it is difficult to explain how past and present renal comorbidities affect renal TRAEs, TRAEs in the lungs and endocrine system were observed in a higher proportion of patients with vs without past or present pulmonary disease and thyroid disease, respectively, suggesting that pulmonary and thyroid comorbidities might be risk factors for pulmonary and endocrine TRAEs, respectively. These data from patients with comorbidities in this PMS may be useful to call attention to TRAEs of nivolumab in clinical practice.

On the contrary, patients with a good condition, such as no peritoneal metastasis, Glasgow prognostic score of 0, CRP level < 1, and prior surgical treatment for G/GEJ cancer, are likely to experience significantly more TRAEs. Moreover, higher incidences of TRAEs in patients with no peritoneal metastasis and a Glasgow prognostic score of 0 and CRP level < 1 were associated with an increased exposure period of nivolumab. The multivariate analysis adjusted by the treatment period identified the absence of peritoneal metastasis and any past or present comorbidities as factors associated with TRAEs. Both the absence of peritoneal metastasis and a Glasgow prognostic score of 0 are well-known favorable prognostic factors. It is considered that the active immune status in patients with a good condition may be related to TRAEs. About 54–82% of patients who experienced TRAEs of special interest were resolved/resolving by pharmacotherapy such as corticosteroids and hormone replacement or by observation only. As some TRAEs were not resolved, leading to sequelae and/or death, early diagnosis and optimal treatment for TRAEs are very important in clinical practice.

Tumor response among the response evaluation set (CR, 1.2%; PR, 10.1%; SD, 27.1%; and PD, 58.3%) and the 6-month survival rate (52.0%) were consistent with those reported in the ATTRACTION-2 trial [4]. Reportedly, the occurrence of TRAEs was associated with better efficacy of nivolumab in patients with metastatic melanoma [7] and advanced G/GEJ cancer [8]. In the CheckMate 141 study in patients with recurrent or metastatic head and neck cancer receiving nivolumab, the median OS (14.3 vs 5.2 months; hazard ratio, 0.10; 95% confidence interval, 0.03–0.39) and 2-year OS rates (31.3 vs 0%) were higher in patients with TRAEs, particularly skin-related disorders, than in those without TRAEs [9]. Similarly, the ORR (18.5 vs 6.7%) was higher in patients who reported TRAEs than in those without TRAEs in this PMS study. When ORR was compared according to patient background characteristics in this PMS, ORR increased with increasing age from ≥ 50 years. However, in the ATTRACTION-2 trial, there was no clear relationship between age and response. Thus, the reason for a higher ORR in the elderly remains unknown. Biological and/or immunological background, such as tumor mutation burden, might be confounding in this PMS study. Furthermore, the limited number of patients in the categories by age may have impacted data interpretation. It is expected that biomarkers for efficacy and TRAEs will be established in the future.

As is the nature of PMS studies, clinical information could not be collected as precisely as in the clinical trials, and the observation period was rather short. Additionally, no biomarker analysis was conducted. A comparison between subgroups could be affected by factors that were not collected or evaluated. Also, multivariate analysis could not be performed for factors such as tumor location and histological differentiation because some patients were counted repeatedly for each tumor location and differentiation. However, this study could cover a wide population of patients with unresectable advanced or recurrent G/GEJ cancer receiving nivolumab, and the obtained results can provide useful information reflecting the real-world scenario.

## Conclusions

The results of this PMS study showed that the safety and effectiveness of nivolumab as salvage (after ≥ 2 lines) therapy in Japanese patients with unresectable advanced or recurrent G/GEJ cancer was consistent with that observed in the phase 3 ATTRACTION-2 study. No new safety concerns were reported over the 6-month observation period.

## Supplementary Information

Below is the link to the electronic supplementary material.

Supplementary file1 (DOCX 155 KB)
