# Supplementary material for: Real-world safety and effectiveness of nivolumab in Japanese patients with unresectable advanced or recurrent gastric/gastroesophageal junction cancer that has progressed after chemotherapy: a postmarketing surveillance study
Source: Gastric Cancer. 2021 Sep 28;25(1):245–53. doi: 10.1007/s10120-021-01244-y (PMC8732939; doi:10.1007/s10120-021-01244-y)
Supplement: Supplementary file 1 — Supplementary file1 (DOCX 155 KB) [file 10120_2021_1244_MOESM1_ESM.docx]

**Supplementary data**

**Supplementary Fig. 1** Patient disposition

CRF, case report form; RECIST, Response Evaluation Criteria in Solid Tumors.

**Supplementary Table 1** Incidence of treatment-related adverse events categorized by patient background factors (safety analysis set)

| **Patient background factors** | **N** | **Incidence of treatment-related adverse events** | | |
| --- | --- | --- | --- | --- |
|  | **N=650** | **n (%)** | **95% CI^a^** | **p value** |
| **Age (years)** |  |  |  |  |
| 20 to <30 | 5 | 1 (20.0) | 0.51-71.64 | 0.1368^b^ |
| 30 to <40 | 10 | 4 (40.0) | 12.16-73.76 |  |
| 40 to <50 | 32 | 8 (25.0) | 11.46-43.40 |  |
| 50 to <60 | 52 | 16 (30.8) | 18.72-45.10 |  |
| 60 to <70 | 242 | 68 (28.1) | 22.53-34.21 |  |
| 70 to <80 | 254 | 90 (35.4) | 29.56-41.66 |  |
| ≥80 | 55 | 18 (32.7) | 20.68-46.71 |  |
| **Sex** | | | | |
| Male | 474 | 156 (32.9) | 28.69-37.34 | 0.2543^c^ |
| Female | 176 | 49 (27.8) | 21.36-35.08 |  |
| **ECOG PS** |  |  |  |  |
| 0 | 265 | 93 (35.1) | 29.36-41.17 | 0.0672^b^ |
| 1 | 320 | 96 (30.0) | 25.03-35.35 |  |
| 2 | 55 | 15 (27.3) | 16.14-40.96 |  |
| 3 | 10 | 1 (10.0) | 0.25-44.50 |  |
| **BMI (kg/m^2^) (n=643)** | | | | |
| <18.5 | 196 | 52 (26.5) | 20.49-33.29 | 0.1495^b^ |
| ≥18.5 to <25 | 404 | 140 (34.7) | 30.02-39.52 |  |
| ≥25 | 43 | 12 (27.9) | 15.33-43.67 |  |
| Unknown | 7 | 1 (14.3) | – |  |
| **Smoking** |  |  |  |  |
| Smoker | 338 | 111 (32.8) | 27.86-38.13 | 0.7171^c^ |
| Nonsmoker | 238 | 74 (31.1) | 25.27-37.39 |  |
| Unknown | 74 | 20 (27.0) | – |  |
| **Drinking** |  |  |  |  |
| None | 281 | 88 (31.3) | 25.94-37.09 | 0.9704^d^ |
| Currently drinks | 106 | 32 (30.2) | 21.65-39.87 |  |
| Currently does not drink | 149 | 47 (31.5) | 24.18-39.65 |  |
| Unknown | 114 | 38 (33.3) | – |  |
| **Any Past or present comorbidity** | | | | |
| No | 221 | 56 (25.3) | 19.74-31.61 | 0.0160^c^* |
| Yes | 428 | 149 (34.8) | 30.30-39.54 |  |
| Unknown | 1 | 0 (0.0) | – |  |
| **Past or present renal disease** | | | | |
| No | 610 | 186 (30.5) | 26.86-34.32 | 0.0212^c^* |
| Yes | 39 | 19 (48.7) | 32.42-65.22 |  |
| Unknown | 1 | 0 (0.0) | – |  |
| **Past or present pulmonary disease** | | | | |
| No | 591 | 177 (29.9) | 26.28-33.82 | 0.0071^c^* |
| Yes | 58 | 28 (48.3) | 34.95-61.78 |  |
| Unknown | 1 | 0 (0.0) | – |  |
| **Past or present thyroid disease** | | | | |
| No | 607 | 184 (30.3) | 26.68-34.14 | 0.0225^c^* |
| Yes | 41 | 20 (48.8) | 32.88-64.87 |  |
| Unknown | 2 | 1 (50.0) | – |  |
| **Past or present hepatic disease** | | | | |
| No | 593 | 183 (30.9) | 27.16-34.75 | 0.2286^c^ |
| Yes | 56 | 22 (39.3) | 26.50-53.25 |  |
| Unknown | 1 | 0 (0.0) | – |  |
| **Pastor present cardiovascular disease** | | | | |
| No | 595 | 187 (31.4) | 27.71-35.33 | 0.8797^c^ |
| Yes | 55 | 18 (32.7) | 20.68-46.71 |  |
| **Past or present autoimmune disease** | | | | |
| No | 629 | 199 (31.6) | 28.02-35.43 | 1.0000^c^ |
| Yes | 20 | 6 (30.0) | 11.89-54.28 |  |
| Unknown | 1 | 0 (0.0) | – |  |
| **Location^f^** | | | | |
| GEJ | 94 | 30 (31.9) | 22.67-42.33 | – |
| Stomach | 543 | 171 (31.5) | 27.60-35.58 |  |
| Others | 8 | 3 (37.5) | 8.52-75.51 |  |
| Unknown | 7 | 2 (28.6) | – |  |
| **Disease status** | | | | |
| New onset | 369 | 108 (29.3) | 24.67-34.20 | 0.1728^c^ |
| Recurrence | 281 | 97 (34.5) | 28.97-40.40 |  |
| **Histological type^f^** | | | | |
| Well differentiated | 343 | 117 (34.1) | 29.10-39.39 | – |
| Poorly differentiated | 385 | 114 (29.6) | 25.09-34.45 |  |
| Others | 7 | 3 (42.9) | 9.90-81.59 |  |
| Unknown | 4 | 1 (25.0) | – |  |
| ***HER2* status** | | | | |
| Negative | 469 | 149 (31.8) | 27.58-36.20 | 0.7469^c^ |
| Positive | 127 | 38 (29.9) | 22.12-38.68 |  |
| Unknown | 54 | 18 (33.3) | – |  |
| **Lymph node metastasis (other than regional lymph nodes)** | | | | |
| Absent | 334 | 108 (32.3) | 27.35-37.64 | 0.6734^c^ |
| Present | 316 | 97 (30.7) | 25.65-36.10 |  |
| **Liver metastasis** | | | | |
| Absent | 444 | 141 (31.8) | 27.45-36.31 | 0.9278^c^ |
| Present | 206 | 64 (31.1) | 24.82-37.87 |  |
| **Lung metastasis** | | | | |
| Absent | 559 | 173 (31.0) | 27.14-34.96 | 0.4656^c^ |
| Present | 91 | 32 (35.2) | 25.44-45.88 |  |
| **Peritoneal metastasis** | | | | |
| Absent | 299 | 107 (35.8) | 30.35-41.51 | 0.0344^c^* |
| Present | 351 | 98 (27.9) | 23.29-32.93 |  |
| **Amount of ascites** |  |  |  |  |
| None or small | 528 | 174 (33.0) | 28.96-37.15 | 0.0766^c^ |
| Large | 115 | 28 (24.4) | 16.83-33.23 |  |
| Unknown | 7 | 3 (42.9) |  |  |
| **Other metastatic sites** | | | | |
| Absent | 583 | 179 (30.7) | 26.98-34.62 | 0.2110^c^ |
| Present | 67 | 26 (38.8) | 27.14-51.50 |  |
| **Albumin level^e^ (g/dL; n=642)** | | | | |
| <3.5 | 377 | 111 (29.4) | 24.89-34.33 | 0.1304^b^ |
| ≥3.5 | 265 | 93 (35.1) | 29.36-41.17 |  |
| Unknown | 8 | 1 (12.5) | – |  |
| **CRP level^e^ (mg/dL; n=626)** | | | | |
| <0.5 | 349 | 115 (33.0) | 28.04-38.16 | 0.2752^b^ |
| ≥0.5 | 277 | 80 (28.9) | 23.61-34.61 |  |
| Unknown | 24 | 10 (41.7) | – |  |
| **CRP level^e^ (mg/dL; n=626)** | | | | |
| <1.0 | 431 | 145 (33.6) | 29.19-38.32 | 0.0455^b^* |
| ≥1.0 | 195 | 50 (25.6) | 19.67-32.37 |  |
| Unknown | 24 | 10 (41.7) | – |  |
| **CRP level^e^ (mg/dL; n=626)** | | | | |
| <5.0 | 582 | 185 (31.8) | 28.02-35.74 | 0.2114^b^ |
| ≥5.0 | 44 | 10 (22.7) | 11.47-37.84 |  |
| Unknown | 24 | 10 (41.7) | – |  |
| **Glasgow prognostic score^e^** | | | | |
| 0 | 211 | 75 (35.6) | 29.09-42.41 | 0.0298^c^* |
| 1 | 258 | 82 (31.8) | 26.15-37.84 |  |
| 2 | 151 | 37 (24.5) | 17.88-32.16 |  |
| Unknown | 30 | 11 (36.7) |  |  |
| **NLR^e^ (n=641)** | | | | |
| <2.5 | 321 | 111 (34.6) | 29.38-40.06 | 0.1130^b^ |
| ≥2.5 | 320 | 92 (28.8) | 23.85-34.05 |  |
| Unknown | 9 | 2 (22.2) | – |  |
| **NLR^e^ (n=641)** | | | | |
| <5.0 | 542 | 181 (33.4) | 29.43-37.54 | 0.0281^b^* |
| ≥5.0 | 99 | 22 (22.2) | 14.48-31.69 |  |
| Unknown | 9 | 2 (22.2) | – |  |
| **Prior treatment for G/GEJ cancer (radiation therapy)** | | | | |
| Absent | 610 | 195 (32.0) | 28.28-35.83 | 0.4823^c^ |
| Present | 40 | 10 (25.0) | 12.69-41.20 |  |
| **Prior treatment for G/GEJ cancer (surgery)** | | | | |
| Absent | 266 | 72 (27.1) | 21.82-32.83 | 0.0482^c^* |
| Present | 384 | 133 (34.6) | 29.88-39.63 |  |
| **Prior treatment for G/GEJ cancer (adjuvant chemotherapy)** | | | | |
| Absent | 407 | 125 (30.7) | 26.26-35.45 | 0.5994^c^ |
| Present | 240 | 79 (32.9) | 27.01-39.25 |  |
| Unknown | 3 | 1 (33.3) | – |  |
| **Antitumor drugs contained in adjuvant chemotherapy** | | | | |
| S1 | 217 | 73 (33.6) | 27.39-40.35 |  |
| Capecitabine | 18 | 4 (22.2) | 6.41-47.64 |  |
| Cisplatin | 42 | 11 (26.2) | 13.86-42.04 |  |
| Oxaliplatin | 49 | 9 (18.4) | 8.76-32.02 |  |
| Others | 28 | 9 (32.1) | 15.88-52.35 |  |
| **Number of lines of prior chemotherapy** | | | | |
| 0 | 2 | 1 (50.0) | 1.26-98.74 | 0.0281^b^ |
| 1 | 12 | 2 (16.7) | 2.09-48.41 |  |
| 2 | 464 | 136 (29.3) | 25.20-33.68 |  |
| 3 | 132 | 53 (40.2) | 31.72-49.04 |  |
| ≥4 | 40 | 13 (32.5) | 18.57-49.13 |  |
| **Details of the prior treatment (cases of drug therapy excluding adjuvant chemotherapy)** | | | | |
| S1 + cisplatin + paclitaxel | 1 | 0 (0.0) | 0.00-97.50 | – |
| S1 + cisplatin | 3 | 1 (33.3) | 0.84-90.57 |  |
| S1 + oxaliplatin + paclitaxel | 1 | 0 (0.0) | 0.00-97.50 |  |
| S1 + oxaliplatin | 8 | 3 (37.5) | 8.52-75.51 |  |
| S1 + docetaxel | 1 | 1 (100.0) | 2.50-100.00 |  |
| S1 + paclitaxel | 3 | 1 (33.3) | 0.84-90.57 |  |
| S1 + nab-paclitaxel | 1 | 0 (0.0) | 0.00-97.50 |  |
| S1 | 6 | 1 (16.7) | 0.42-64.12 |  |
| Capecitabine + cisplatin | 1 | 0 (0.0) | 0.00-97.50 |  |
| Capecitabine + oxaliplatin + trastuzumab | 2 | 1 (50.0) | 1.26-98.74 |  |
| Capecitabine + oxaliplatin + ramucirumab + paclitaxel | 1 | 1 (100.0) | 2.50-100.00 |  |
| Capecitabine + oxaliplatin | 11 | 4 (36.4) | 10.93-69.21 |  |
| Capecitabine + trastuzumab | 2 | 0 (0.0) | 0.00-84.19 |  |
| Capecitabine | 2 | 0 (0.0) | 0.00-84.19 |  |
| 5FU + oxaliplatin | 4 | 1 (25.0) | 0.63-80.59 |  |
| 5FU | 2 | 0 (0.0) | 0.00-84.19 |  |
| Cisplatin + irinotecan | 6 | 3 (50.0) | 11.81-88.19 |  |
| Oxaliplatin + ramucirumab | 1 | 0 (0.0) | 0.00-97.50 |  |
| Trastuzumab + paclitaxel | 2 | 0 (0.0) | 0.00-84.19 |  |
| Trastuzumab + irinotecan | 1 | 1 (100.0) | 2.50-100.00 |  |
| Ramucirumab + paclitaxel + irinotecan | 1 | 1 (100.0) | 2.50-100.00 |  |
| Ramucirumab + paclitaxel | 368 | 123 (33.4) | 28.62-38.50 |  |
| Ramucirumab + nab-paclitaxel | 49 | 13 (26.5) | 14.95-41.08 |  |
| Ramucirumab + irinotecan | 1 | 0 (0.0) | 0.00-97.50 |  |
| Ramucirumab | 20 | 6 (30.0) | 11.89-54.28 |  |
| Docetaxel | 5 | 3 (60.0) | 14.66-94.73 |  |
| Paclitaxel | 39 | 9 (23.1) | 11.13-39.33 |  |
| Nab-paclitaxel | 21 | 2 (9.5) | 1.17-30.38 |  |
| Irinotecan | 80 | 26 (32.5) | 22.45-43.89 |  |
| Others | 12 | 5 (41.7) | 15.17-72.33 |  |
| **Number of doses (times)** | | | | |
| 1-4 | 325 | 91 (28.0) | 23.18-33.22 | 0.0337^b^* |
| 5-8 | 167 | 52 (31.1) | 24.21-38.75 |  |
| 9-12 | 75 | 35 (46.7) | 35.05-58.55 |  |
| 13-16 | 83 | 27 (32.5) | 22.65-43.70 |  |
| **Dosage** |  |  |  |  |
| 3 mg/kg throughout the study | 460 | 134 (29.1) | 25.02-33.52 | 0.1179^c^ |
| 240 mg/body throughout the study | 69 | 27 (39.1) | 27.60-51.63 |  |
| Changed to 240 mg/body during the study | 77 | 31 (40.3) | 29.23-52.06 |  |
| Others | 44 | 13 (29.6) | 16.76-45.20 |  |
| **Concomitant drug (antitumor drug)** | | | | |
| Absent | 634 | 200 (31.6) | 27.94-35.32 | 1.0000^c^ |
| Present | 16 | 5 (31.3) | 11.02-58.66 |  |
| **Concomitant drug (vaccine)** | | | | |
| Absent | 642 | 203 (31.6) | 28.04-35.37 | 1.0000^c^ |
| Present | 8 | 2 (25.0) | 3.19-65.09 |  |
| **Concomitant treatment (surgical therapy)** | | | | |
| Absent | 636 | 201 (31.6) | 28.00-35.37 | 1.0000^c^ |
| Present | 14 | 4 (28.6) | 8.39-58.10 |  |
| **Concomitant treatment (radiation therapy)** | | | | |
| Absent | 635 | 202 (31.8) | 28.20-35.59 | 0.4106^c^ |
| Present | 15 | 3 (20.0) | 4.33-48.09 |  |

*p<0.05 was considered to be statistically significant.

^a^Calculated by a method based on the exact method.

^b^Statistical test used: Wilcoxon rank sum test.

^c^Statistical test used: Fisher’s exact test.

^d^Statistical test used: chi-square test.

^e^Within 2 weeks before initiating nivolumab

^f^Statistical analysis could not be performed as the total number exceeds the number of the whole population because more than one category for tumor location and histological differentiation were checked in the case report form in some patients.

5FU, 5-fluorouracil; BMI, body mass index; CI, confidence interval; CRP, C-reactive protein; ECOG PS, Eastern Cooperative Oncology Group performance status; GEJ, gastroesophageal junction; *HER2*, human epidermal growth factor receptor 2; NLR, neutrophil to lymphocyte ratio; S1, Tegafur/gimeracil/oteracil.; G/GEJ, Gastric/gastroesophageal junction

**Supplementary Table 2** Past or present medical history categorized by TRAEs

|  | | **Medical history** | | | | **Medical history (kidney)** | | | | **Medical history (lungs)** | | | | **Medical history (thyroid)** | | | |
| --- | --- | --- | --- | --- | --- | --- | --- | --- | --- | --- | --- | --- | --- | --- | --- | --- | --- |
|  |  | **Yes** | | **No** | | **Yes** | | **No** | | **Yes** | | **No** | | **Yes** | | **No** | |
| **No. of evaluated cases** | | 428 | | 221 | | 39 | | 610 | | 58 | | 591 | | 41 | | 607 | |
| **No. of cases with adverse reactions** | | 149 | | 56 | | 19 | | 186 | | 28 | | 177 | | 20 | | 184 | |
| **Incidence of adverse reactions (%)** | | 34.8 | | 25.3 | | 48.7 | | 30.5 | | 48.3 | | 30.0 | | 48.8 | | 30.3 | |
| **Types of adverse reactions (SOC・PT)** | | **n** | **(%)** | **n** | **(%)** | **n** | **(%)** | **n** | **(%)** | **n** | **(%)** | **n** | **(%)** | **n** | **(%)** | **n** | **(%)** |
| **Infections and infestations** | | 8 | (1.9) | 2 | (0.9) | 2 | (5.1) | 8 | (1.3) | 1 | (1.7) | 9 | (1.5) | 1 | (2.4) | 9 | (1.5) |
|  | Appendicitis perforated | 0 | - | 1 | (0.5) | 0 | - | 1 | (0.2) | 0 | - | 1 | (0.2) | 0 | - | 1 | (0.2) |
|  | Gastrointestinal candidiasis | 1 | (0.2) | 0 | - | 0 | - | 1 | (0.2) | 0 | - | 1 | (0.2) | 0 | - | 1 | (0.2) |
|  | Herpes zoster | 1 | (0.2) | 0 | - | 1 | (2.6) | 0 | - | 0 | - | 1 | (0.2) | 0 | - | 1 | (0.2) |
|  | Paronychia | 1 | (0.2) | 0 | - | 0 | - | 1 | (0.2) | 0 | - | 1 | (0.2) | 0 | - | 1 | (0.2) |
|  | Pneumonia | 2 | (0.5) | 1 | (0.5) | 1 | (2.6) | 2 | (0.3) | 1 | (1.7) | 2 | (0.3) | 0 | - | 3 | (0.5) |
|  | Pyelonephritis | 1 | (0.2) | 0 | - | 0 | - | 1 | (0.2) | 0 | - | 1 | (0.2) | 1 | (2.4) | 0 | - |
|  | Bone abscess | 1 | (0.2) | 0 | - | 0 | - | 1 | (0.2) | 0 | - | 1 | (0.2) | 0 | - | 1 | (0.2) |
|  | *Pneumocystis jirovecii* pneumonia | 1 | (0.2) | 0 | - | 0 | - | 1 | (0.2) | 0 | - | 1 | (0.2) | 0 | - | 1 | (0.2) |
| **Neoplasms benign, malignant and unspecified (incl cysts and polyps)** | | 1 | (0.2) | 1 | (0.5) | 0 | - | 2 | (0.3) | 0 | - | 2 | (0.3) | 0 | - | 2 | (0.3) |
|  | Malignant pericarditis | 0 | - | 1 | (0.5) | 0 | - | 1 | (0.2) | 0 | - | 1 | (0.2) | 0 | - | 1 | (0.2) |
|  | Pyogenic granuloma | 1 | (0.2) | 0 | - | 0 | - | 1 | (0.2) | 0 | - | 1 | (0.2) | 0 | - | 1 | (0.2) |
| **Blood and lymphatic system disorders** | | 6 | (1.4) | 3 | (1.4) | 1 | (2.6) | 8 | (1.3) | 1 | (1.7) | 8 | (1.4) | 0 | - | 9 | (1.5) |
|  | Anaemia | 6 | (1.4) | 3 | (1.4) | 1 | (2.6) | 8 | (1.3) | 1 | (1.7) | 8 | (1.4) | 0 | - | 9 | (1.5) |
| **Endocrine disorders** | | 31 | (7.2) | 8 | (3.6) | 4 | (10.3) | 35 | (5.7) | 8 | (13.8) | 31 | (5.2) | **11** | **(26.8)** | **27** | **(4.4)** |
|  | Adrenal insufficiency | 2 | (0.5) | 1 | (0.5) | 0 | - | 3 | (0.5) | 1 | (1.7) | 2 | (0.3) | **1** | **(2.4)** | **2** | **(0.3)** |
|  | Hyperthyroidism | 3 | (0.7 | 1 | (0.5) | 0 | - | 4 | (0.7) | 1 | (1.7) | 3 | (0.5) | **1** | **(2.4)** | **3** | **(0.5)** |
|  | Hypopituitarism | 0 | - | 1 | (0.5) | 0 | - | 1 | (0.2) | 0 | - | 1 | (0.2) | **0** | **-** | **1** | **(0.2)** |
|  | Hypothyroidism | 22 | (5.1) | 5 | (2.3) | 3 | (7.7) | 24 | (3.9) | 5 | (8.6) | 22 | (3.7) | **7** | **(17.1)** | **20** | **(3.3)** |
|  | Secondary adrenocortical insufficiency | 1 | (0.2) | 0 | - | 0 | - | 1 | (0.2) | 1 | (1.7) | 0 | - | **0** | **-** | **1** | **(0.2)** |
|  | Thyroid disorder | 1 | (0.2) | 0 | - | 0 | - | 1 | (0.2) | 0 | - | 1 | (0.2) | **0** | **-** | **1** | **(0.2)** |
|  | Thyroiditis | 1 | (0.2) | 1 | (0.5) | 0 | - | 2 | (0.3) | 0 | - | 2 | (0.3) | **1** | **(2.4)** | **1** | **(0.2)** |
|  | Inappropriate antidiuretic hormone secretion | 1 | (0.2) | 0 | - | 0 | - | 1 | (0.2) | 0 | - | 1 | (0.2) | **0** | **-** | **0** | **-** |
|  | Hypophysitis | 1 | (0.2) | 0 | - | 1 | (2.6) | 0 | - | 1 | (1.7) | 0 | - | **1** | **(2.4)** | **0** | **-** |
|  | ACTH (adrenocorticotropic hormone) deficiency | 1 | (0.2) | 0 | - | 0 | - | 1 | (0.2) | 0 | - | 1 | (0.2) | **1** | **(2.4)** | **0** | **-** |
| **Metabolism and nutrition disorders** | | 23 | (5.4) | 5 | (2.3) | 4 | (10.3) | 24 | (3.9) | 3 | (5.2) | 25 | (4.2) | 3 | (7.3) | 25 | (4.1) |
|  | Dehydration | 0 | - | 1 | (0.5) | 0 | - | 1 | (0.2) | 0 | - | 1 | (0.2) | 0 | - | 1 | (0.2) |
|  | Diabetic ketoacidosis | 1 | (0.2) | 0 | - | 0 | - | 1 | (0.2) | 0 | - | 1 | (0.2) | 0 | - | 1 | (0.2) |
|  | Hyperkalaemia | 1 | (0.2) | 1 | (0.5) | 0 | - | 2 | (0.3) | 0 | - | 2 | (0.3) | 1 | (2.4) | 1 | (0.2) |
|  | Hyperuricaemia | 1 | (0.2) | 0 | - | 0 | - | 1 | (0.2) | 0 | - | 1 | (0.2) | 0 | - | 1 | (0.2) |
|  | Hypoalbuminaemia | 2 | (0.5) | 0 | - | 0 | - | 2 | (0.3) | 0 | - | 2 | (0.3) | 0 | - | 2 | (0.3) |
|  | Hypokalaemia | 1 | (0.2) | 0 | - | 0 | - | 1 | (0.2) | 0 | - | 1 | (0.2) | 0 | - | 1 | (0.2) |
|  | Hyponatraemia | 3 | (0.7) | 0 | - | 2 | (5.1) | 1 | (0.2) | 1 | (1.7) | 2 | (0.3) | 1 | (2.4) | 2 | (0.3) |
|  | Decreased appetite | 15 | (3.5) | 4 | (1.8) | 2 | (5.1) | 17 | (2.8) | 2 | (3.4) | 17 | (2.9) | 2 | (4.9) | 17 | (2.8) |
|  | Fulminant type 1 diabetes | 2 | (0.5) | 0 | - | 0 | - | 2 | (0.3) | 0 | - | 2 | (0.3 | 0 | - | 2 | (0.3) |
| **Nervous system disorders** | | 12 | (2.8) | 1 | (0.5) | 0 | - | 13 | (2.1) | 0 | - | 13 | (2.2) | 0 | - | 13 | (2.1) |
|  | Cerebral infarction | 4 | (0.9) | 0 | - | 0 | - | 4 | (0.7) | 0 | - | 4 | (0.7) | 0 | - | 4 | (0.7) |
|  | Dizziness | 1 | (0.2) | 0 | - | 0 | - | 1 | (0.2) | 0 | - | 1 | (0.2) | 0 | - | 1 | (0.2) |
|  | Taste deficiency | 1 | (0.2) | 0 | - | 0 | - | 1 | (0.2) | 0 | - | 1 | (0.2) | 0 | - | 1 | (0.2) |
|  | Haemorrhage intracranial | 1 | (0.2) | 0 | - | 0 | - | 1 | (0.2) | 0 | - | 1 | (0.2) | 0 | - | 1 | (0.2) |
|  | Headache | 1 | (0.2) | 0 | - | 0 | - | 1 | (0.2) | 0 | - | 1 | (0.2) | 0 | - | 1 | (0.2) |
|  | Hypoaesthesia | 1 | (0.2) | 0 | - | 0 | - | 1 | (0.2) | 0 | - | 1 | (0.2) | 0 | - | 1 | (0.2) |
|  | Myasthenia gravis | 1 | (0.2) | 0 | - | 0 | - | 1 | (0.2) | 0 | - | 1 | (0.2) | 0 | - | 1 | (0.2) |
|  | Nervous system disorder | 1 | (0.2) | 0 | - | 0 | - | 1 | (0.2) | 0 | - | 1 | (0.2) | 0 | - | 1 | (0.2) |
|  | Recurrent laryngeal nerve palsy | 0 | - | 1 | (0.5) | 0 | - | 1 | (0.2) | 0 | - | 1 | (0.2) | 0 | - | 1 | (0.2) |
|  | Peripheral sensory neuropathy | 1 | (0.2) | 0 | - | 0 | - | 1 | (0.2) | 0 | - | 1 | (0.2) | 0 | - | 1 | (0.2) |
|  | Hypogeusia | 1 | (0.2) | 0 | - | 0 | - | 1 | (0.2) | 0 | - | 1 | (0.2) | 0 | - | 1 | (0.2) |
| **Eye disorders** | | 1 | (0.2) | 0 | - | 0 | - | 1 | (0.2) | 0 | - | 1 | (0.2) | 0 | - | 1 | (0.2) |
|  | Vision blurred | 1 | (0.2) | 0 | - | 0 | - | 1 | (0.2) | 0 | - | 1 | (0.2) | 0 | - | 1 | (0.2) |
| **Ear and labyrinth disorders** | | 1 | (0.2) | 0 | - | 0 | - | 1 | (0.2) | 0 | - | 1 | (0.2) | 1 | (2.4) | 0 | - |
|  | Vertigo | 1 | (0.2) | 0 | - | 0 | - | 1 | (0.2) | 0 | - | 1 | (0.2) | 1 | (2.4) | 0 | - |
| **Vascular disorders** | | 1 | (0.2) | 0 | - | 1 | (2.6) | 0 | - | 0 | - | 1 | (0.2) | 0 | - | 1 | (0.2) |
|  | Embolism venous | 1 | (0.2) | 0 | - | 1 | (2.6) | 0 | - | 0 | - | 1 | (0.2) | 0 | - | 1 | (0.2) |
| **Respiratory, thoracic and mediastinal disorders** | | 18 | (4.2) | 3 | (1.4) | 0 | - | 21 | (3.4) | **5** | **(8.6)** | **16** | **(2.7)** | 2 | (4.9) | 19 | (3.1) |
|  | Cough | 2 | (0.5) | 0 | - | 0 | - | 2 | (0.3) | **0** | **-** | **2** | **(0.3)** | 0 | - | 2 | (0.3) |
|  | Dyspnoea | 1 | (0.2) | 0 | - | 0 | - | 1 | (0.2) | **1** | **(1.7)** | **0** | **-** | 0 | - | 1 | (0.2) |
|  | Interstitial lung disease | 8 | (1.9) | 2 | (0.9) | 0 | - | 10 | (1.6) | **3** | **(5.2)** | **7** | **(1.2)** | 1 | (2.4) | 9 | (1.5) |
|  | Lung disorder | 1 | (0.2) | 0 | - | 0 | - | 1 | (0.2) | **1** | **(1.7)** | **0** | **-** | 1 | (2.4) | 0 | - |
|  | Pleural effusion | 1 | (0.2) | 1 | (0.5) | 0 | - | 2 | (0.3) | **0** | **-** | **2** | **(0.3)** | 0 | - | 2 | (0.3) |
|  | Pneumonitis | 4 | (0.9) | 0 | - | 0 | - | 4 | (0.7) | **1** | **(1.7)** | **3** | **(0.5)** | 0 | - | 4 | (0.7) |
|  | Pulmonary embolism | 1 | (0.2) | 0 | - | 0 | - | 1 | (0.2) | **0** | **-** | **1** | **(0.2)** | 0 | - | 1 | (0.2) |
|  | Organising pneumonia | 1 | (0.2) | 0 | - | 0 | - | 1 | (0.2) | **0** | **-** | **1** | **(0.2)** | 0 | - | 1 | (0.2) |
| **Gastrointestinal disorders** | | 27 | (6.3) | 19 | (8.6) | 5 | (12.8) | 41 | (6.7) | 6 | (10.3) | 40 | (6.8) | 4 | (9.8) | 42 | (6.9) |
|  | Cheilitis | 1 | (0.2) | 0 | - | 0 | - | 1 | (0.2) | 0 | - | 1 | (0.2) | 0 | - | 1 | (0.2) |
|  | Colitis | 2 | (0.5) | 0 | - | 1 | (2.6) | 1 | (0.2) | 1 | (1.7) | 1 | (0.2) | 1 | (2.4) | 1 | (0.2) |
|  | Constipation | 2 | (0.5) | 1 | (0.5) | 0 | - | 3 | (0.5) | 0 | - | 3 | (0.5) | 0 | - | 3 | (0.5) |
|  | Diarrhoea | 14 | (3.3) | 10 | (4.5) | 4 | (10.3) | 20 | (3.3) | 3 | (5.2) | 21 | (3.6) | 2 | (4.9) | 22 | (3.6) |
|  | Dry mouth | 1 | (0.2) | 0 | - | 0 | - | 1 | (0.2) | 0 | - | 1 | (0.2) | 0 | - | 1 | (0.2) |
|  | Gastric distension | 0 | - | 1 | (0.5) | 0 | - | 1 | (0.2) | 0 | - | 1 | (0.2) | 0 | - | 1 | (0.2) |
|  | Blood stool excretion | 0 | - | 1 | (0.5) | 0 | - | 1 | (0.2) | 0 | - | 1 | (0.2) | 0 | - | 1 | (0.2) |
|  | Paralytic ileus | 0 | - | 1 | (0.5) | 0 | - | 1 | (0.2) | 0 | - | 1 | (0.2) | 0 | - | 1 | (0.2) |
|  | Intestinal ischaemia | 1 | (0.2) | 0 | - | 0 | - | 1 | (0.2) | 0 | - | 1 | (0. 2) | 0 | - | 1 | (0. 2) |
|  | Nausea | 4 | (0.9) | 2 | (0.9) | 0 | - | 6 | (1.0) | 1 | (1.7) | 5 | (0.8) | 0 | - | 6 | (1.0) |
|  | Oesophagitis | 0 | - | 1 | (0.5) | 0 | - | 1 | (0.2) | 0 | - | 1 | (0.2) | 0 | - | 1 | (0.2) |
|  | Oral pain | 0 | - | 1 | (0.5) | 0 | - | 1 | (0.2) | 0 | - | 1 | (0.2) | 0 | - | 1 | (0.2) |
|  | Stomatitis | 4 | (0.9) | 4 | (1.8) | 0 | - | 8 | (1.3) | 0 | - | 8 | (1.4) | 0 | - | 8 | (1.3) |
|  | Upper gastrointestinal haemorrhage | 1 | (0.2) | 0 | - | 0 | - | 1 | (0.2) | 0 | - | 1 | (0.2) | 0 | - | 1 | (0.2) |
|  | Vomiting | 4 | (0.9) | 0 | - | 1 | (2.6) | 3 | (0.5) | 2 | (3.4) | 2 | (0.3) | 1 | (2.4) | 3 | (0.5) |
|  | Gastrointestinal oedema | 0 | - | 1 | (0.5) | 0 | - | 1 | (0.2) | 0 | - | 1 | (0.2) | 0 | - | 1 | (0.2) |
|  | Diverticular perforation | 1 | (0.2) | 0 | - | 0 | - | 1 | (0.2) | 0 | - | 1 | (0.2) | 0 | - | 1 | (0.2) |
|  | Loose stool | 1 | (0.2) | 0 | - | 0 | - | 1 | (0.2) | 1 | (1.7) | 0 | - | 1 | (2.4) | 0 | - |
|  | Dyschezia | 1 | (0.2) | 0 | - | 0 | - | 1 | (0.2) | 0 | - | 1 | (0.2) | 0 | - | 1 | (0.2) |
| **Hepatobiliary disorders** | | 6 | (1.4) | 4 | (1.8) | 1 | (2.6) | 9 | (1.5) | 2 | (3.4) | 8 | (1.4) | 1 | (2.4) | 9 | (1.5) |
|  | Cholangitis | 1 | (0.2) | 0 | - | 0 | - | 1 | (0.2) | 1 | (1.7) | 0 | - | 0 | - | 1 | (0.2) |
|  | Hepatic function abnormal | 2 | (0.5) | 2 | (0.9) | 1 | (2.6) | 3 | (0.5) | 1 | (1.7) | 3 | (0.5) | 1 | (2.4) | 3 | (0.5) |
|  | Liver disorder | 2 | (0.5) | 2 | (0.9) | 0 | - | 4 | (0.7) | 0 | - | 4 | (0.7) | 0 | - | 4 | (0.7) |
|  | Disease of the hepatobiliary system | 1 | (0.2) | 0 | - | 0 | - | 1 | (0.2) | 0 | - | 1 | (0.2) | 0 | - | 1 | (0.2) |
| **Skin and subcutaneous tissue disorders** | | 31 | (7.2) | 7 | (3.2) | 4 | (10.3) | 34 | (5.6) | 3 | (5.2) | 35 | (5.9) | 3 | (7.3) | 35 | (5.8) |
|  | Alopecia | 1 | (0.2) | 0 | - | 0 | - | 1 | (0.2) | 0 | - | 1 | (0.2) | 0 | - | 1 | (0.2) |
|  | Blister | 0 | - | 1 | (0.5) | 0 | - | 1 | (0.2) | 0 | - | 1 | (0.2) | 0 | - | 1 | (0.2) |
|  | Dry skin | 1 | (0.2) | 0 | - | 0 | - | 1 | (0.2) | 1 | (1.7) | 0 | - | 1 | (2.4) | 0 | - |
|  | Eczema | 2 | (0.5) | 0 | - | 0 | - | 2 | (0.3) | 0 | - | 2 | (0.3) | 1 | (2.4) | 1 | (0.2) |
|  | Palmar-plantar erythrodysesthesia syndrome | 1 | (0.2) | 0 | - | 0 | - | 1 | (0.2) | 0 | - | 1 | (0.2) | 0 | - | 1 | (0.2) |
|  | Pruritus | 7 | (1.6) | 3 | (1.4) | 2 | (5.1) | 8 | (1.3) | 1 | (1.7) | 9 | (1.5) | 0 | - | 10 | (1.6) |
|  | Psoriasis | 1 | (0.2) | 0 | - | 0 | - | 1 | (0.2) | 0 | - | 1 | (0.2) | 1 | (2.4) | 0 | - |
|  | Pustular psoriasis | 1 | (0.2) | 0 | - | 0 | - | 1 | (0.2) | 0 | - | 1 | (0.2) | 0 | - | 1 | (0.2) |
|  | Rash | 14 | (3.3) | 2 | (0.9) | 2 | (5.1) | 14 | (2.3) | 1 | (1.7) | 15 | (2.5) | 0 | - | 16 | (2.6) |
|  | Rash maculo-papular | 4 | (0.9) | 0 | - | 1 | (2.6) | 3 | (0.5) | 0 | - | 4 | (0.7) | 0 | - | 4 | (0.7) |
|  | Rash pruritic | 1 | (0.2) | 0 | - | 0 | - | 1 | (0.2) | 0 | - | 1 | (0.2) | 0 | - | 1 | (0.2) |
|  | Skin disorder | 1 | (0.2) | 1 | (0.5) | 1 | (2.6) | 1 | (0.2) | 0 | - | 2 | (0.3) | 0 | - | 2 | (0.3) |
| **Musculoskeletal and connective tissue disorders** | | 9 | (2.1) | 1 | (0.5) | 0 | - | 10 | (1.6) | 2 | (3.4) | 8 | (1.4) | 0 | - | 10 | (1.6) |
|  | Arthralgia | 4 | (0.9) | 0 | - | 0 | - | 4 | (0.7) | 1 | (1.7) | 3 | (0.5) | 0 | - | 4 | (0.7) |
|  | Arthritis | 1 | (0.2) | 0 | - | 0 | - | 1 | (0.2) | 0 | - | 1 | (0.2) | 0 | - | 1 | (0.2) |
|  | Muscle spasms | 1 | (0.2) | 0 | - | 0 | - | 1 | (0.2) | 0 | - | 1 | (0.2) | 0 | - | 1 | (0.2) |
|  | Myalgia | 4 | (0.9) | 0 | - | 0 | - | 4 | (0.7) | 2 | (3.4) | 2 | (0.3) | 0 | - | 4 | (0.7) |
|  | Polymyalgia rheumatica | 0 | - | 1 | (0.5) | 0 | - | 1 | (0.2) | 0 | - | 1 | (0.2) | 0 | - | 1 | (0.2) |
|  | Rhabdomyolysis | 1 | (0.2) | 0 | - | 0 | - | 1 | (0.2) | 0 | - | 1 | (0.2) | 0 | - | 1 | (0.2) |
| **Renal and urinary disorders** | | 2 | (0.5) | 1 | (0.5) | **0** | **-** | **3** | **(0.5)** | 0 | - | 3 | (0.5) | 0 | - | 3 | (0.5) |
|  | Glycosuria | 1 | (0.2) | 0 | - | **0** | **-** | **1** | **(0.2)** | 0 | - | 1 | (0.2) | 0 | - | 1 | (0.2) |
|  | Renal disorder | 1 | (0.2) | 0 | - | **0** | **-** | **1** | **(0.2)** | 0 | - | 1 | (0.2) | 0 | - | 1 | (0.2) |
|  | Renal impairment | 0 | - | 1 | (0.5) | **0** | **-** | **1** | **(0.2)** | 0 | - | 1 | (0.2) | 0 | - | 1 | (0.2) |
| **Reproductive system and breast disorders** | | 1 | (0.2) | 0 | - | 0 | - | 1 | (0.2) | 1 | (1.7) | 0 | - | 0 | - | 1 | (0.2) |
|  | Gynaecomastia | 1 | (0.2) | 0 | - | 0 | - | 1 | (0.2) | 1 | (1.7) | 0 | - | 0 | - | 1 | (0.2) |
| **General disorders and administration site conditions** | | 26 | (6.1) | 7 | (3.2) | 2 | (5.1) | 31 | (5.1) | 1 | (1.7) | 32 | (5.4) | 1 | (2.4) | 32 | (5.3) |
|  | Fatigue | 8 | (1.9) | 1 | (0.5) | 1 | (2.6) | 8 | (1.3) | 0 | - | 9 | (1.5) | 0 | - | 9 | (1.5) |
|  | Injection site reaction | 1 | (0.2) | 0 | - | 0 | - | 1 | (0.2) | 0 | - | 1 | (0.2) | 0 | - | 1 | (0.2) |
|  | Malaise | 11 | (2.6) | 4 | (1.8) | 1 | (2.6) | 14 | (2.3) | 1 | (1.7) | 14 | (2.4) | 1 | (2.4) | 14 | (2.3) |
|  | Oedema peripheral | 1 | (0.2) | 0 | - | 0 | - | 1 | (0.2) | 0 | - | 1 | (0.2) | 0 | - | 1 | (0.2) |
|  | Pyrexia | 6 | (1.4) | 2 | (0.9) | 0 | - | 8 | (1.3) | 0 | - | 8 | (1.4) | 0 | - | 8 | (1.3) |
|  | Ulcer | 0 | - | 1 | (0.5) | 0 | - | 1 | (0.2) | 0 | - | 1 | (0.2) | 0 | - | 1 | (0.2) |
|  | Infusion site extravasation | 1 | (0.2) | 0 | - | 0 | - | 1 | (0.2) | 0 | - | 1 | (0.2) | 0 | - | 1 | (0.2) |
|  | Multiple organ dysfunction syndrome | 1 | (0.2) | 0 | - | 0 | - | 1 | (0.2) | 0 | - | 1 | (0.2) | 0 | - | 1 | (0.2) |
| **Investigations** | | 27 | (6.3) | 12 | (5.4) | 7 | (17.9) | 32 | (5.2) | 7 | (12.1) | 32 | (5.4) | 4 | (9.8) | 35 | (5.8) |
|  | Alanine aminotransferase increased | 6 | (1.4) | 1 | (0.5) | 2 | (5.1) | 5 | (0.8) | 3 | (5.2) | 4 | (0.7) | 1 | (2.4) | 6 | (1.0) |
|  | Aspartate aminotransferase increased | 7 | (1.6) | 0 | - | 3 | (7.7) | 4 | (0.7) | 3 | (5.2) | 4 | (0.7) | 1 | (2.4) | 6 | (1.0) |
|  | Blood bilirubin increased | 2 | (0.5) | 1 | (0.5) | 1 | (2.6) | 2 | (0.3) | 1 | (1.7) | 2 | (0.3) | 0 | - | 3 | (0.5) |
|  | Blood corticotropin decreased | 2 | (0.5) | 1 | (0.5) | 0 | - | 3 | (0.5) | 2 | (3.4) | 1 | (0.2) | 0 | - | 3 | (0.5) |
|  | Blood creatine phosphokinase increased | 3 | (0.7) | 2 | (0.9) | 0 | - | 5 | (0.8) | 0 | - | 5 | (0.8) | 1 | (2.4) | 4 | (0.7) |
|  | Blood creatinine increased | 3 | (0.7) | 2 | (0.9) | 2 | (5.1) | 3 | (0.5) | 0 | - | 5 | (0.8) | 0 | - | 5 | (0.8) |
|  | Blood glucose increased | 1 | (0.2) | 0 | - | 0 | - | 1 | (0.2) | 0 | - | 1 | (0.2) | 0 | - | 1 | (0.2) |
|  | Blood lactate dehydrogenase increased | 1 | (0.2) | 0 | - | 1 | (2.6) | 0 | - | 0 | - | 1 | (0.2) | 0 | - | 1 | (0.2) |
|  | Blood pressure decreased | 1 | (0.2) | 0 | - | 1 | (2.6) | 0 | - | 1 | (1.7) | 0 | - | 1 | (2.4) | 0 | - |
|  | Blood sodium decreased | 1 | (0.2) | 0 | - | 0 | - | 1 | (0.2) | 0 | - | 1 | (0.2) | 0 | - | 1 | (0.2) |
|  | Blood thyroid-stimulating hormone decreased | 1 | (0.2) | 1 | (0.5) | 0 | - | 2 | (0.3) | 1 | (1.7) | 1 | (0.2) | 0 | - | 2 | (0.3) |
|  | Blood thyroid-stimulating hormone increased | 1 | (0.2) | 2 | (0.9) | 0 | - | 3 | (0.5) | 0 | - | 3 | (0.5) | 0 | - | 3 | (0.5) |
|  | C-reactive protein increased | 1 | (0.2) | 0 | - | 0 | - | 1 | (0.2) | 0 | - | 1 | (0.2) | 0 | - | 1 | (0.2) |
|  | Gamma-glutamyltransferase increased | 2 | (0.5) | 0 | - | 1 | (2.6) | 1 | (0.2) | 0 | - | 2 | (0.3) | 0 | - | 2 | (0.3) |
|  | Glucose urine present | 1 | (0.2) | 0 | - | 0 | - | 1 | (0.2) | 0 | - | 1 | (0.2 | 0 | - | 1 | (0.2) |
|  | Blood urine present | 1 | (0.2) | 0 | - | 1 | (2.6) | 0 | - | 0 | - | 1 | (0.2) | 0 | - | 1 | (0.2) |
|  | Neutrophil count decreased | 2 | (0.5) | 0 | - | 0 | - | 2 | (0.3) | 0 | - | 2 | (0.3) | 0 | - | 2 | (0.3) |
|  | Platelet count decreased | 3 | (0.7) | 1 | (0.5) | 1 | (2.6) | 3 | (0.5) | 0 | - | 4 | (0.7) | 0 | - | 4 | (0.7) |
|  | Thyroid function test abnormal | 0 | - | 1 | (0.5) | 0 | - | 1 | (0.2) | 0 | - | 1 | (0.2) | 0 | - | 1 | (0.2) |
|  | Vitamin B12 decreased | 0 | - | 1 | (0.5) | 0 | - | 1 | (0.2) | 0 | - | 1 | (0.2) | 0 | - | 1 | (0.2) |
|  | White blood cell count decreased | 3 | (0.7) | 1 | (0.5) | 1 | (2.6) | 3 | (0.5) | 0 | - | 4 | (0.7) | 0 | - | 4 | (0.7) |
|  | Blood alkaline phosphatase increased | 4 | (0.9) | 0 | - | 2 | (5.1) | 2 | (0.3) | 2 | (3.4) | 2 | (0.3) | 0 | - | 4 | (0.7) |
|  | Hepatic enzyme increased | 1 | (0.2) | 0 | - | 0 | - | 1 | (0.2) | 0 | - | 1 | (0.2) | 1 | (2.4) | 0 | - |
|  | Thyroid hormone decreased | 1 | (0.2) | 0 | - | 0 | - | 1 | (0.2) | 0 | - | 1 | (0.2) | 1 | (2.4) | 0 | - |
| **Injury, poisoning, and procedural complications** | | 2 | (0.5) | 2 | (0.9) | 1 | (2.6) | 3 | (0.5) | 0 | - | 4 | (0.7) | 1 | (2.4) | 3 | (0.5) |
|  | Infusion related reaction | 2 | (0.5) | 2 | (0.9) | 1 | (2.6) | 3 | (0.5) | 0 | - | 4 | (0.7) | 1 | (2.4) | 3 | (0.5) |

AE, adverse event; PT, preferred term; SOC, system organ class; TRAE, treatment-related adverse event.

**Supplementary Table 3** Summary of patients with past or present renal disease

| **Number of patients with past or present renal disease: 39/650 (6%)** | | | |
| --- | --- | --- | --- |
| **Total number of renal medical histories (preferred term): 42** | | | |
| **System organ class** | **n (%)** | **Type of medical history (preferred term)** | **N (%)** |
| Infections and infestations | 1 (0.2) | Pyelonephritis | 1 (0.2) |
| Neoplasms benign, malignant and unspecified (incl cysts and polyps) | 2 (0.3) | Renal cancer | 1 (0.2) |
|  |  | Renal cell cancer | 1 (0.2) |
| Renal and urinary disorders | 36 (5.5) | Chronic glomerulonephritis | 1 (0.2) |
|  |  | Membranous glomerulonephritis | 1 (0.2) |
|  |  | Haematuria | 1 (0.2) |
|  |  | Hydronephrosis | 13 (2.0) |
|  |  | Nephrolithiasis | 2 (0.3) |
|  |  | Nephrotic syndrome | 4 (0.6) |
|  |  | Proteinuria | 2 (0.3) |
|  |  | Diabetic nephropathy | 2 (0.3) |
|  |  | Renal impairment | 6 (0.9) |
|  |  | Chronic kidney disease | 5 (0.8) |
| Investigations | 2 (0.3) | Blood creatinine increased | 2 (0.3) |

Types of medical history were classified using MedDRA/J v22.1 and listed by system organ class and preferred term.

MedDRA/J, Medical Dictionary for Regulatory Activities Japanese version.

**Supplementary Table 4** Summary of patients with past or present pulmonary disease

| **Number of patients with past or present pulmonary disease: 58/650 (8.9%)** | | | |
| --- | --- | --- | --- |
| **Total number of pulmonary medical histories (preferred term): 64** | | | |
| **System organ class** | **n (%)** | **Type of medical history (preferred term)** | **n (%)** |
| Infections and infestations | 17 (2.6) | Pneumonia | 12 (1.9) |
|  |  | Pulmonary tuberculosis | 3 (0.5) |
|  |  | Tuberculosis | 1 (0.2) |
|  |  | Infectious pleural effusion | 1 (0.2) |
| Neoplasms benign, malignant and unspecified (incl cysts and polyps) | 9 (1.4) | Lung adenocarcinoma | 1 (0.2) |
|  |  | Metastases to lung | 2 (0.3) |
|  |  | Small cell lung cancer | 1 (0.2) |
|  |  | Lung neoplasm malignant | 5 (0.8) |
| Respiratory, thoracic and mediastinal disorders | 33 (5.1) | Asthma | 8 (1.2) |
|  |  | Bronchiectasis | 1 (0.2) |
|  |  | Bronchitis chronic | 1 (0.2) |
|  |  | Chronic obstructive pulmonary disease | 5 (0.8) |
|  |  | Emphysema | 5 (0.8) |
|  |  | Hyperventilation | 1 (0.2) |
|  |  | Interstitial lung disease | 6 (0.9) |
|  |  | Lung disorder | 1 (0.2) |
|  |  | Pleural effusion | 1 (0.2) |
|  |  | Pneumothorax | 2 (0.3) |
|  |  | Pulmonary (lung) thrombosis | 2 (0.3) |
|  |  | Pulmonary infarction | 1 (0.2) |
|  |  | Respiratory failure | 1 (0.2) |
|  |  | Organising pneumonia | 1 (0.2) |
|  |  | Idiopathic interstitial pneumonia | 1 (0.2) |
| Injury, poisoning and procedural complications | 1 (0.2) | Asbestosis | 1 (0.2) |

Types of medical history were classified using MedDRA/J v22.1 and listed by system organ class and preferred term.

MedDRA/J, Medical Dictionary for Regulatory Activities Japanese version.

**Supplementary Table 5** Summary of patients with past or present thyroid disease

| **Number of patients with past or present thyroid disease: 41/650 (6.3%)** | | | |
| --- | --- | --- | --- |
| **Total number of thyroid medical histories (preferred term): 41** | | | |
| **System organ class** | **n (%)** | **Type of medical history (preferred term)** | **n (%)** |
| Neoplasms benign, malignant and unspecified (including cysts and polyps) | 3 (0.5) | Thyroid cancer | 3 (0.5) |
| Endocrine disorders | 38 (5.8) | Basedow's disease | 1 (0.2) |
|  |  | Goitre | 2 (0.3) |
|  |  | Hyperthyroidism | 2 (0.3) |
|  |  | Hypothyroidism | 28 (4.3) |
|  |  | Thyroiditis | 1 (0.2) |
|  |  | Chronic thyroiditis | 1 (0.2) |
|  |  | Autoimmune thyroiditis | 3 (0.5) |

Types of medical history were classified using MedDRA/J v22.1 and listed by system organ class and preferred term.

MedDRA/J, Medical Dictionary for Regulatory Activities Japanese version.

**Supplementary Table** **6** Tumor response/effectiveness categorized by patient background characteristics (response evaluation set: excluding patients whose tumor response was NE; patients whose evaluation method complied with RECIST v1.1)

| **Patient background factors** | **Number of patients** | **Number of improved patients^a^** | **ORR (%)** | **95% CI^b^** | **p value** |
| --- | --- | --- | --- | --- | --- |
| **Overall** | 499 | 58 | 11.6 | 8.95-14.77 |  |
| **Age (1) (years)** | | | | | |
| 20 to <30 | 5 | 0 | 0.0 | 0.00-52.18 | 0.0020^d^* |
| 30 to <40 | 6 | 0 | 0.0 | 0.00-45.93 |  |
| 40 to <50 | 23 | 0 | 0.0 | 0.00-14.82 |  |
| 50 to <60 | 38 | 2 | 5.3 | 0.64-17.75 |  |
| 60 to <70 | 184 | 20 | 10.9 | 6.77-16.29 |  |
| 70 to <80 | 203 | 26 | 12.8 | 8.54-18.20 |  |
| ≥80 | 40 | 10 | 25.0 | 12.69-41.20 |  |
| **Age (2) (years)** |  |  |  |  |  |
| <65 | 132 | 7 | 5.3 | 2.16-10.62 | 0.0083 ^d^* |
| ≥65 | 367 | 51 | 13.9 | 10.52-17.86 |  |
| **Age (3) (years)** |  |  |  |  |  |
| <75 | 371 | 34 | 9.2 | 6.43-12.57 | 0.0036 ^d^* |
| ≥75 | 128 | 24 | 18.8 | 12.40-26.60 |  |
| **Sex** |  |  |  |  |  |
| Male | 369 | 46 | 12.5 | 9.27-16.28 | 0.4260^c^ |
| Female | 130 | 12 | 9.2 | 4.86-15.57 |  |
| **ECOG PS** |  |  |  |  |  |
| 0 | 231 | 24 | 10.4 | 6.77-15.06 | 0.5900^d^ |
| 1 | 232 | 31 | 13.4 | 9.26-18.43 |  |
| 2 | 34 | 3 | 8.8 | 1.86-23.68 |  |
| 3 | 2 | 0 | 0.0 | 0.00-84.19 |  |
| **BMI (kg/m^2^) (n=493)** |  |  |  |  |  |
| <18.5 | 128 | 11 | 8.6 | 4.37-14.86 | 0.0784^d^ |
| ≥18.5 to <25 | 331 | 40 | 12.1 | 8.78-16.09 |  |
| ≥25 | 34 | 7 | 20.6 | 8.70-37.90 |  |
| Unknown | 6 | 0 | 0.0 | – |  |
| **Smoking** |  |  |  |  |  |
| Smoker | 253 | 34 | 13.4 | 9.49-18.27 | 0.1815^c^ |
| Nonsmoker | 198 | 18 | 9.1 | 5.48-13.99 |  |
| Unknown | 48 | 6 | 12.5 | – |  |
| **Drinking** |  |  |  |  |  |
| None | 211 | 25 | 11.9 | 7.82-16.99 | 0.9752^e^ |
| Currently drinks | 95 | 12 | 12.6 | 6.70-21.03 |  |
| Currently not drink | 112 | 14 | 12.5 | 7.01-20.08 |  |
| Unknown | 81 | 7 | 8.6 | – |  |
| **Any past or present comorbidity** | | | | | |
| No | 171 | 17 | 9.9 | 5.90-15.44 | 0.4628^c^ |
| Yes | 327 | 41 | 12.5 | 9.15-16.62 |  |
| Unknown | 1 | 0 | 0.0 | – |  |
| **Past or present renal disease** | | | | | |
| No | 471 | 56 | 11.9 | 9.11-15.16 | 0.7570^c^ |
| Yes | 27 | 2 | 7.4 | 0.91-24.29 |  |
| Unknown | 1 | 0 | 0.0 | – |  |
| **Past or present pulmonary disease** | | | | | |
| No | 457 | 50 | 10.9 | 8.23-14.17 | 0.1229^c^ |
| Yes | 41 | 8 | 19.5 | 8.82-34.87 |  |
| Unknown | 1 | 0 | 0.0 | – |  |
| **Past or present thyroid disease** | | | | | |
| No | 460 | 52 | 11.3 | 8.56-14.56 | 0.4202^c^ |
| Yes | 37 | 6 | 16.2 | 6.19-32.01 |  |
| Unknown | 2 | 0 | 0.0 | – |  |
| **Past or present hepatic disease** | | | | | |
| No | 460 | 54 | 11.7 | 8.94-15.04 | 1.0000^c^ |
| Yes | 38 | 4 | 10.5 | 2.94-24.80 |  |
| Unknown | 1 | 0 | 0.0 | – |  |
| **Past or present cardiovascular disease** | | | | | |
| No | 456 | 55 | 12.1 | 9.22-15.41 | 0.4559^c^ |
| Yes | 43 | 3 | 7.0 | 1.46-19.06 |  |
| **Past or present autoimmune disease** | | |  |  |  |
| No | 485 | 56 | 11.6 | 8.84-14.73 | 0.6551^c^ |
| Yes | 13 | 2 | 15.4 | 1.92-45.45 |  |
| Unknown | 1 | 0 | 0.0 | – |  |
| **Location**^g^ | | | | | |
| GEJ | 76 | 6 | 7.9 | 2.95-16.40 | – |
| Stomach | 413 | 52 | 12.6 | 9.55-16.18 |  |
| Others | 6 | 0 | 0.0 | 0.00-45.93 |  |
| Unknown | 5 | 0 | 0.0 | – |  |
| **Disease status** |  |  |  |  |  |
| New onset | 287 | 29 | 10.1 | 6.87-14.19 | 0.2584^c^ |
| Recurrence | 212 | 29 | 13.7 | 9.36-19.05 |  |
| **Histological type**^g,^ | | | | | |
| Well differentiated | 268 | 32 | 11.9 | 8.31-16.44 | – |
| Poorly differentiated | 296 | 31 | 10.5 | 7.23-14.54 |  |
| Others | 4 | 2 | 50.0 | 6.76-93.24 |  |
| Unknown | 3 | 0 | 0.0 | – |  |
| ***HER2* status** |  |  |  |  |  |
| Negative | 361 | 45 | 12.5 | 9.24-16.32 | 0.5909^c^ |
| Positive | 94 | 9 | 9.6 | 4.47-17.40 |  |
| Unknown | 44 | 4 | 9.1 | - |  |
| **Lymph node metastasis (other than regional lymph nodes)** | | | | | |
| Absent | 248 | 27 | 10.9 | 7.30-15.44 | 0.6759^c^ |
| Present | 251 | 31 | 12.4 | 8.55-17.07 |  |
| **Liver metastasis** | | | | | |
| Absent | 335 | 38 | 11.3 | 8.15-15.24 | 0.7682^c^ |
| Present | 164 | 20 | 12.2 | 7.61-18.20 |  |
| **Lung metastasis** | | | | | |
| Absent | 430 | 53 | 12.3 | 9.37-15.81 | 0.3105^c^ |
| Present | 69 | 5 | 7.3 | 2.39-16.11 |  |
| **Peritoneal metastasis** | |  |  |  |  |
| Absent | 249 | 34 | 13.7 | 9.65-18.56 | 0.1653^c^ |
| Present | 250 | 24 | 9.6 | 6.25-13.95 |  |
| **Amount of ascites** |  |  |  |  |  |
| None or small | 421 | 50 | 11.9 | 8.94-15.36 | 1.0000^c^ |
| Large | 74 | 8 | 10.8 | 4.78-20.20 |  |
| Unknown | 4 | 0 | 0 | - |  |
| **Other metastatic sites** | | | | | |
| Absent | 447 | 51 | 11.4 | 8.61-14.73 | 0.6482^c^ |
| Present | 52 | 7 | 13.5 | 5.59-25.79 |  |
| **Albumin level**^f^ **(g/dL; n=493)** | | | | | |
| <3.5 | 271 | 27 | 10.0 | 6.67-14.16 | 0.2207^d^ |
| ≥3.5 | 222 | 30 | 13.5 | 9.31-18.73 |  |
| Unknown | 6 | 1 | 16.7 | – |  |
| **CRP level**^f^ **(mg/dL; n=480)** | | | | | |
| <0.5 | 288 | 32 | 11.1 | 7.73-15.32 | 0.5272^d^ |
| ≥0.5 | 192 | 25 | 13.0 | 8.61-18.62 |  |
| Unknown | 19 | 1 | 5.3 | – |  |
| **CRP level**^f^ **(mg/dL; n=480)** | | | | | |
| <1.0 | 349 | 44 | 12.6 | 9.31-16.55 | 0.4190^d^ |
| ≥1.0 | 131 | 13 | 9.9 | 5.39-16.37 |  |
| Unknown | 19 | 1 | 5.3 | - |  |
| **CRP level**^f^ **(mg/dL; n=480)** | | | | | |
| <5.0 | 454 | 54 | 11.9 | 9.06-15.23 | 0.09576^d^ |
| ≥5.0 | 26 | 3 | 11.5 | 2.45-30.15 |  |
| Unknown | 19 | 1 | 5.3 |  |  |
| **Glasgow prognostic score**^f^ | | | | | |
| 0 | 352 | 44 | 12.5 | 9.23-16.42 | 0.5190^d^ |
| 1 | 32 | 3 | 9.4 | 1.98-25.02 |  |
| 2 | 95 | 10 | 10.5 | 5.16-18.51 |  |
| Unknown | 20 | 1 | 5.0 | - |  |
| **NLR**^f^ **(n=493)** | | | | | |
| <2.5 | 265 | 32 | 12.1 | 8.41-16.62 | 0.8180^d^ |
| ≥2.5 | 228 | 26 | 11.4 | 7.59-16.26 |  |
| Unknown | 6 | 0 | 0 |  |  |
| **NLR**^f^ **(n=493)** | | | | | |
| <5.0 | 431 | 50 | 11.6 | 8.73-15.01 | 0.7669^d^ |
| ≥5.0 | 62 | 8 | 12.9 | 5.74-23.85 |  |
| Unknown | 6 | 0 | 0 |  |  |
| **Prior treatment for G/GEJ cancer (radiation therapy)** | | | | | |
| Absent | 471 | 56 | 11.9 | 9.11-15.16 | 0.7595^c^ |
| Present | 28 | 2 | 7.1 | 0.88-23.50 |  |
| **Prior treatment for G/GEJ cancer (surgery)** | | | | | |
| Absent | 207 | 24 | 11.6 | 7.57-16.76 | 1.0000^c^ |
| Present | 292 | 34 | 11.6 | 8.20-15.89 |  |
| **Prior treatment for G/GEJ cancer (adjuvant chemotherapy)** | | | | | |
| Absent | 314 | 34 | 10.8 | 7.62-14.80 | 0.4705^c^ |
| Present | 183 | 24 | 13.1 | 8.59-18.88 |  |
| Unknown | 2 | 0 | 0.0 | – |  |
| **Antitumor drugs contained in adjuvant chemotherapy** | | | | | |
| S1 | 168 | 19 | 11.3 | 6.95-17.10 | – |
| Capecitabine | 13 | 4 | 30.8 | 9.09-61.43 |  |
| Cisplatin | 27 | 3 | 11.1 | 2.35-29.16 |  |
| Oxaliplatin | 36 | 6 | 16.7 | 6.37-32.81 |  |
| Others | 18 | 2 | 11.1 | 1.38-34.71 |  |
| **Number of lines of prior chemotherapy** | | | | | |
| 1 | 0 | 0 | - | - | 0.9671^d^ |
| 2 | 365 | 43 | 11.8 | 8.66-15.54 |  |
| 3 | 104 | 10 | 9.6 | 4.71-16.97 |  |
| ≥4 | 30 | 5 | 16.7 | 5.64-34.72 |  |
| **Details of the prior treatment line^d^ (cases of drug therapy excluding adjuvant chemotherapy)** | | | | | |
| S1 + cisplatin | 3 | 0 | 0.0 | 0.00-70.76 | – |
| S1 + oxaliplatin + paclitaxel | 1 | 0 | 0.0 | 0.00-97.50 |  |
| S1 + oxaliplatin | 6 | 2 | 33.3 | 4.33-77.72 |  |
| S1 + docetaxel | 1 | 1 | 100.0 | 2.50-100.00 |  |
| S1 + paclitaxel | 2 | 1 | 50.0 | 1.26-98.74 |  |
| S1 + nab-paclitaxel | 1 | 0 | 0.0 | 0.00-97.50 |  |
| S1 | 4 | 0 | 0.0 | 0.00-60.24 |  |
| Capecitabine + cisplatin | 1 | 0 | 0.0 | 0.00-97.50 |  |
| Capecitabine + oxaliplatin + trastuzumab | 2 | 1 | 50.0 | 1.26-98.74 |  |
| Capecitabine + oxaliplatin + ramucirumab + paclitaxel | 1 | 1 | 100.0 | 2.50-100.00 |  |
| Capecitabine + oxaliplatin | 9 | 0 | 0.0 | 0.00-33.63 |  |
| Capecitabine + trastuzumab | 1 | 0 | 0.0 | 0.00-97.50 |  |
| Capecitabine | 2 | 0 | 0.0 | 0.00-84.19 |  |
| 5FU + oxaliplatin | 2 | 0 | 0.0 | 0.00-84.19 |  |
| 5FU | 2 | 0 | 0.0 | 0.00-84.19 |  |
| Cisplatin + irinotecan | 6 | 0 | 0.0 | 0.00-45.93 |  |
| Oxaliplatin + ramucirumab | 1 | 0 | 0.0 | 0.00-97.50 |  |
| Trastuzumab + paclitaxel | 1 | 0 | 0.0 | 0.00-97.50 |  |
| Trastuzumab + irinotecan | 1 | 1 | 100.0 | 2.50-100.00 |  |
| Ramucirumab + paclitaxel + irinotecan | 1 | 0 | 0.0 | 0.00-97.50 |  |
| Ramucirumab + paclitaxel | 280 | 28 | 10.0 | 6.75-14.13 |  |
| Ramucirumab + nab-paclitaxel | 38 | 4 | 10.5 | 2.94-24.80 |  |
| Ramucirumab + irinotecan | 1 | 1 | 100.0 | 2.50-100.00 |  |
| Ramucirumab | 17 | 6 | 35.3 | 14.21-61.67 |  |
| Docetaxel | 3 | 0 | 0.0 | 0.00-70.76 |  |
| Paclitaxel | 28 | 5 | 17.9 | 6.06-36.89 |  |
| Nab-paclitaxel | 16 | 0 | 0.0 | 0.00-20.59 |  |
| Irinotecan | 66 | 7 | 10.6 | 4.37-20.64 |  |
| Others | 8 | 1 | 12.5 | 0.32-52.65 |  |
| **Number of doses (times)** | | | | | |
| 1-4 | 211 | 4 | 1.9 | 0.52-4.78 | <0.0001^d^ |
| 5-8 | 152 | 10 | 6.6 | 3.20-11.77 |  |
| 9-12 | 67 | 13 | 19.4 | 10.76-30.89 |  |
| 13-16 | 69 | 31 | 44.9 | 32.92-57.38 |  |
| **Dosage** |  |  |  |  |  |
| 3 mg/kg throughout the study | 342 | 33 | 9.7 | 6.74-13.28 | 0.0888^c^ |
| 240 mg/body throughout the study | 58 | 10 | 17.2 | 8.59-29.43 |  |
| Changed to 240 mg/body during the study | 64 | 12 | 18.8 | 10.08-30.46 |  |
| Others | 35 | 3 | 8.6 | 1.80-23.06 |  |
| **Concomitant drug (antitumor drug)** | | |  |  |  |
| Absent | 486 | 56 | 11.5 | 8.82-14.70 | 0.6547^c^ |
| Present | 13 | 2 | 15.4 | 1.92-45.45 |  |
| **Concomitant drug (vaccine)** | | |  |  |  |
| Absent | 491 | 56 | 11.4 | 8.73-14.55 | 0.2357^c^ |
| Present | 8 | 2 | 25.0 | 3.19-65.09 |  |
| **Concomitant treatment (surgical therapy)** | | | |  |  |
| Absent | 487 | 56 | 11.5 | 8.80-14.67 | 0.6390^c^ |
| Present | 12 | 2 | 16.7 | 2.09-48.41 |  |
| **Concomitant treatment (radiation therapy)** | | | |  |  |
| Absent | 489 | 58 | 11.9 | 9.13 | 0.6144^c^ |
| Present | 10 | 0 | - | 0.0 |  |

^a^The number of cases in which tumor response was CR or PR.

^b^Calculated by a method based on the exact method.

*p<0.05 was considered to be statistically significant.

^c^Statistical test used: Fisher’s exact test.

^d^Statistical test used: Wilcoxon rank sum test.

^e^Statistical test used: Chi-square test

^f^Within 2 weeks before initiating nivolumab

^g^Statistical analysis could not be performed as the total number exceeds the number of the whole population because more than one category for tumor location and histological differentiation were checked in the case report form in some patients.

5FU, 5-fluorouracil; BMI, body mass index; CI, confidence interval; CR, complete response; CRP, C-reactive protein; ECOG PS, Eastern Cooperative Oncology Group performance status; GEJ, gastroesophageal junction; *HER2*, human epidermal growth factor receptor 2; NE, not evaluable; NLR, neutrophil to lymphocyte ratio; ORR, objective response rate; PR, partial response; RECIST, Response Evaluation Criteria in Solid Tumors; S1, Tegafur/gimeracil/oteracil.; G/GEJ, Gastric/gastroesophageal junction

.
